# High-quality genome assembly and comparative analysis reveal extensive genomic variation in Talaromyces marneffei

**DOI:** 10.1099/mgen.0.001400

**Published:** 2025-04-28

**Authors:** Jinxia Luo, Jingyuan Bian, Michaela Murillo, Pak-Ting Hau, Yi Feng, Eddie Chung-Ting Chau, Yuyao Yan, Laam-Ching Ng, Ayesha S. K. Parsha, Gilman Kit-Hang Siu, Franklin Wang-Ngai Chow, Qing Xiong

**Affiliations:** 1 Department of Health Technology and Informatics, Faculty of Health and Social Science, The Hong Kong Polytechnic University, Hong Kong, PR China

**Keywords:** comparative genomics, genomic structural variation, long terminal repeat (LTR) retrotransposons, *Talaromyces marneffei*

## Abstract

*Talaromyces marneffei* is a dimorphic fungus that transitions from a filamentous form at 25 °C to a pathogenic yeast form at 37 °C, demonstrating pathogenicity mostly in immunocompromised individuals, such as those with human immunodeficiency virus/AIDS. Though it is one of the most severe infectious fungi in Southeast Asia, the lack of comprehensive genomic analysis has hindered advancement in strain differentiation, diagnosis and treatment. In this study, we assembled a high-quality genome of *T. marneffei* ATCC 18224, resulting in a 28.9 Mb genome distributed across 11 contigs, using third-generation Oxford Nanopore Technologies sequencing reads. Notably, we identified a strain-specific 740-kb segmental duplication in strain ATCC 18224, potentially mediated by inserting a Ty1/*Copia* long terminal repeat (LTR) retrotransposon. This segmental duplication includes various functional genes, with 75 differentially expressed during its dimorphic transition. Comparative genomic analysis revealed large-scale rearrangements in strains PM1 and 11CN-20-091, which were inconsistent with the phylogenomic trees of six *T. marneffei* strains and required further investigation. Additionally, we observed substantial genetic structural variations in LTR retrotransposons, particularly within the Ty1/*Copia* family, including two significant recent expansions in strain ATCC 18224. In summary, the identification and characterization of these extensive genomic structural variations in *T. marneffei* contribute to a deep understanding of its genetic diversity and will facilitate improvements in genotyping, classification and genomic surveillance.

**Impact Statement**

*Talaromyces marneffei*, a thermally dimorphic fungus, is a significant pathogen in Southeast Asia, particularly affecting immunocompromised individuals. Despite its clinical importance, the genetic mechanisms underlying its pathogenicity remain poorly understood. Our study presents a high-quality genome assembly of the *T. marneffei* strain ATCC 18224, revealing a 740-kb segmental duplication specific to this strain. This duplication includes 299 protein-coding genes, enriching molecular functions such as metal ion binding, which may play a role in fungal stress response and adaptation. Comparative genomic analysis across six *T. marneffei* strains uncovered extensive genomic structural variations, including large-scale chromosomal rearrangements and active Ty1/*Copia* retrotransposon expansions. These findings provide critical insights into the genomic evolution and structural diversity of *T. marneffei*, paving the way for improved genotyping, classification and genome surveillance of this medically important fungus. Our work underscores the necessity for further genomic studies to enhance diagnostic and therapeutic strategies against *T. marneffei* infections.

## Data Availability

All data of *T. marneffei* ATCC 18224, including the genomic DNA sequencing data (SRR31006960), transcriptome sequencing data (SRR31006956, SRR31006957, SRR31006958 and SRR31006959) and annotated genome sequences (GCA_046127475.1), are deposited in the NCBI database under BioProject accession PRJNA1163092.

## Introduction

*Talaromyces marneffei* (Segretain, Capponi and Sureau) Samson, N. Yilmaz, Frisvad & Seifert 2011 (=*Penicillium marneffei*) is the most important thermally dimorphic fungus responsible for respiratory, skin and systemic mycoses in Southeast Asia, including regions such as China, Hong Kong, Thailand, Vietnam and Northern India [1]. It can transition from a filamentous form at 25 °C to a pathogenic yeast form at 37 °C within mammalian hosts [2]. Discovered in 1956 [3], only 18 human cases were reported until 1985 [4]. However, with the onset of the HIV pandemic, particularly in Southeast Asia, *T. marneffei* emerged as a significant opportunistic infection with high mortality rates in human immunodeficiency virus (HIV)-positive patients [1]. Approximately 8% of AIDS patients in Hong Kong are infected with *T. marneffei* [5]. In northern Thailand, talaromycosis (penicilliosis) ranks as the third most common AIDS indicator disease, following tuberculosis and cryptococcosis [6].

Clinically, talaromycosis presents as a systemic febrile illness due to intracellular infection of the reticuloendothelial cells by the yeast phase and the associated inflammatory response of the host. Other than HIV-positive individuals, *T. marneffei* infections are observed in immunocompromised patients, such as transplant recipients, patients with systemic lupus erythematosus or anti-IFN-*γ* autoantibodies and patients undergoing corticosteroid therapy or treatment with anti-CD20 monoclonal antibodies/kinase inhibitors [7]. In 2018, *T. marneffei* was ranked second in the list of the world’s top ten most feared fungi [8]. As of 2022, talaromycosis had been reported in over 0.28 million cases across 34 countries with a mortality rate exceeding 30% [9]. Its inclusion in the World Health Organization’s fungal priority list in October 2022 underscores the need for advancing genomic understanding for better diagnostics, treatment and preventive measures.

Despite its clinical significance, the genetic mechanisms and variations underlying the biology and pathogenicity of *T. marneffei* remain inadequately understood till now. Variations among isolates in biochemical features [10,11], gene alterations [9] and even chromosomal numbers [11] underscore the need for a comprehensive genomic investigation to deepen our scientific understanding and inform clinical applications. Currently, five assemblies of *T. marneffei* with contig N50 values exceeding 1 Mb have been published, all derived from human isolates. However, studies on chromosomal rearrangements within *T. marneffei* are still lacking. Notably, the strain ATCC 18224, the only strain not isolated from a human host, has been widely used as a reference genome. Unfortunately, the previously submitted genome assembly (GCA_000001985.1) for strain ATCC 18224 is of bad quality with fragmented contigs and low coverage, raising concerns about the accuracy of comparative genomic analyses based on this resource. These limitations highlight the urgent necessities for a high-quality genome assembly of strain ATCC 18224 and a comprehensive comparative genomic analysis to provide insights into structural variations of pathogenic fungus, which form the aims of this study.

In this study, we applied Oxford Nanopore Technologies (ONT) sequencing to *T. marneffei* ATCC 18224 and developed a high-quality genome assembly. Within this assembly, we identified a strain-specific segmental duplication as long as 740 kb. Further comparative genomic analysis revealed a wide range of genomic structural variations among different strains of *T. marneffei* using whole-genome sequences. Highly diverse and active long terminal repeat (LTR) retrotransposon families were identified to be potential regulators of the structural variations. Our findings have significant implications for understanding the genomic evolution and structural variations of this medically important fungus.

## Methods

### *T. marneffei* strain and culture conditions

*T. marneffei* ATCC 18224 was acquired from the American Type Culture Collection (ATCC, USA) and cultured on Sabouraud dextrose agar (SDA) (BD DIFCO, USA) at 25 °C for 7 days to obtain the filamentous form. Then, conidia of *T. marneffei* ATCC 18224 were harvested using a cotton swab soaked in PBS (Thermo Fisher Scientific, USA) and applied to a new SDA plate to ensure even distribution. Subsequently, the conidia were inoculated into 25 ml of Sabouraud dextrose broth (SDB) (BD DIFCO) and incubated for 5 days, with temperatures set to 25 °C for the filamentous form and 37 °C for the yeast form.

### Genomic DNA extraction and sequencing

*T. marneffei* ATCC 18224 yeast culture in SDB was centrifuged at 4,347 ***g*** for 20 min. The supernatant was removed to obtain the pellet. Four hundred fifty microlitres of Solution MBL from the QIAamp BiOstic Bacteremia DNA Kit (Qiagen, Germany; Cat no. 12240-50) were added to the pellet, and extraction proceeded following the manufacturer’s protocol. The DNA was quantified by the Qubit 2.0 fluorometer (Thermo Fisher Scientific) with dsDNA BR assay kit (Thermo Fisher Scientific).

Libraries were prepared using the Rapid Barcoding Kit (SQK-RBK110.96) from ONT following the manufacturer’s protocol. Genomic sequencing was conducted on a MinION Flow Cell R10 (FLO-MINI106) with the ONT GridION for 28 h.

### Whole-genome assembly

Raw reads were trimmed using NanoFit [12]. Reads with an average read quality score below 8.0 and a length lower than 1,000 bp were discarded. The filtered clean reads were *de novo* assembled using Flye v2.9.3 [13]. Completeness of genome assembly was assessed by Benchmarking Universal Single-Copy Orthologs (BUSCO) v5.4.2 with database eurotiales_odb10 [14]. Other assembly statistics were assessed by QUAST v5.0.2 [15]. Repeat annotation was performed using RepeatMasker v4.0.8 [16]. Repeat masking was conducted using *de novo* prediction with RepeatModeler v2.0.1 [17] and masking with RepeatMasker v4.0.8 [16] (RepBase edition 20181026). For the *de novo* prediction with RepeatModeler v2.0.1, RECON v1.05 [18] and RepeatScout v1.0.6 [19] were utilized to identify repeat families in the genome.

### RNA extraction and sequencing

The RNA of two forms (filamentous at 25 °C and yeast at 37 °C) was extracted using a RiboPure RNA purification kit (Thermo Fisher, USA) following the manufacturer’s recommendations. RNA concentration and purity were measured using NanoDrop (Thermo Scientific, DE, USA). RNA quality was assessed with Agilent 2100 Bioanalyzer (Agilent Technologies, CA, USA). These samples were replicated for each group and were sent for RNA sequencing using Illumina NovaSeq 6000 to generate PE150 reads by Azenta Life Sciences. There are two replicates for each temperature, and the quality control of RNA sequencing data was conducted by Seqkit v2.9.0 [20] (Table S1, available in the online Supplementary Material). The mapping rates were calculated by Hisat2 v2.2.1 [21]. Transcriptome sequencing data of strain PM1 were downloaded from the NCBI database (BioProject accession: PRJNA212740).

### Genome annotation

The strain ATCC 18224 assembly was structurally annotated by MAKER v3.01.03 pipeline [22] and functionally annotated using Funannotate v1.8.13 [23]. The annotated protein sequences of two *T. marneffei* genomes (GCA_009556855.1 and GCA_009650675.1) were used as the protein homologues, and transcripts were generated from our transcriptome data, as the evidence for the genome annotation. Genome annotation was carried out using the MAKER pipeline, incorporating both evidence-based gene annotation and *ab initio* gene prediction. Within the MAKER pipeline, transcriptome assemblies and homologous proteins from publicly available annotated genomes of *T. marneffei* (NCBI GenBank accessions: GCA_009556855.1 and GCA_009650675.1) were used as alignment evidence by Exonerate v2.4.0 [24]. *Ab initio* gene prediction was performed using SNAP (lib v2017-03-01) [25], GeneMark v4.38 [26] and Augustus v3.3.1 [27]. SNAP and Augustus were trained with default parameters based on the results of the initial round of evidence-based annotation, while GeneMark was trained using a self-training method. The completeness of the annotated protein sequences was evaluated using BUSCO v5.4.2 with the eurotiales_odb10 database. Finally, for functional annotation, InterProScan v5.31 [28] was initially used to annotate protein domains, and the Funannotate pipeline employed eggNOG-mapper v2.1.5 [29] for functional annotation.

### Identification of large segmental duplication

The coverage of sequences was calculated using minimap2 v.2.22-r1101 [30] by mapping raw data to the draft ATCC 18224 genome and by mapping raw data (PM1: SRR1514652, 11CN-03–130: SRR8592562, 11CN-20–091: SRR8592561 and GZ8H79: SRR10538955) to genomes accordingly. The synteny analysis of Seq9, Seq8 and Seq2 in the draft genome of ATCC 18224 and the homologous sequences of the other five *T. marneffei* genomes (GCA_000750115.2, GCA_003971505.1, GCA_013122295.1, GCA_009650675.1 and GCF_009556855.1) was conducted and visualized using GenomeSyn [31]. Read alignments were visualized by Integrative Genomics Viewer (IGV) v2.17.2 [32] to confirm that the junctions are well supported by raw reads.

A PCR experiment was conducted to verify that Edge24, a segment linking two parts of the draft sequences, was inserted between Seq8 and Seq2. Forward and reverse primers were designed to the region spanning over Edge24. Two primer pairs were used, respectively, named pair 1 (forward primer: 5′-TGGAATGGGCAAGGTAAATGC-3′, reverse primer: 5′ATCCCATGATTCGACGCTCCT-3′) and pair 2 (forward primer: 5′-GCCTGGAATGGGCAAGGTAAAT-3′, reverse primer: 5′-ATATCAATCCCATGATTCGACGCT-3′). PCR amplifications were performed in 20 µl reactions comprised of 10 µl 2X Phanta Max Master Mix (P515) (Vazyme Biotech, China), 0.5 µl forward primer (10 µM), 0.5 µl reverse primer (10 µM), 8 µl nuclease-free water and 1 µl total DNA isolate of *T. marneffei* ATCC 18224 in yeast form (normalized to 50 ng µl^−1^). For PCR, initial denaturation was conducted at 95 °C for 3 min, followed by 35 cycles of denaturation (95 °C for 15 s), annealing (69.5 °C for 15 s) and extension (72 °C for 7 min). An equal volume of distilled water was used as the negative control in place of DNA. The final extension was held at 72 °C for 5 min. Gel electrophoresis of the PCR products was run on 1.5 % agarose gel.

Relative quantification with quantitative PCR (qPCR) was conducted to determine the ratio of contig TMATCC0008.1 to Seq2, with Seq2 as an internal control. Three primer sets were designed for TMATCC008.1, while two sets were designed for Seq2 (Table S2), all amplifying single-copy exons in TMATCC008.1 and Seq2. Each qPCR reaction was comprised of 10 µl TB Green Premix Ex Taq II (Tli RNaseH Plus) (Takara, Japan), 0.8 µl forward primer (10 µM), 0.8 µl reverse primer (10 µM), 6.4 µl nuclease-free water and 2 µl of *T. marneffei* ATCC 18224 total DNA isolate (normalized to ~50 ng µl^−1^). The final volume per reaction amounted to 20 µl. qPCR was performed on a Roche LightCycler 480 Instrument II (Roche, USA) following the recommended reaction programme stated in the TB Green Premix Ex Taq (Tli RNaseH Plus) product manual. Pre-incubation was performed at 95 °C for 30 s (ramp rate=4.4 °C s^−1^), followed by denaturation (95 °C for 5 s; ramp rate=4.4 °C s^−1^) and then combined annealing and extension (60 °C for 30 s; ramp rate=2.2 °C s^−1^; single acquisition mode) for 50 cycles. Melt curve analysis was conducted by setting the temperature to 95 °C for 5 s (ramp rate=4.4 °C s^−1^) and then 60 °C for 1 min (ramp rate=2.2 °C s^−1^), followed by raising the temperature to 95 °C (ramp rate=0.11 °C s^−1^; continuous acquisition mode; 5 acquisitions/°C). Finally, cooling was performed at 50 °C for 30 s (ramp rate=2.2 °C s^−1^). Ct values were calculated on the Roche LightCycler 480 II software with high confidence.

### Gene enrichment analysis of TMATCC008.1

All the encoded protein sequences in TMATCC008.1 were extracted and mapped to *Saccharomyces cerevisiae* to obtain UniProt accession. Gene Ontology (GO) and KEGG enrichment analyses of these accessions were performed by the online tool DAVID [33] and visualized by SRplot [34]. The linear map of TMATCC008.1 was visualized by Proksee viewer [35]. Coding DNA sequences (CDS) of genes in TMATCC008.1 were extracted using Gffread v0.12.7 [36].

### Differential expression analysis

The expression levels of the genes were then calculated using Salmon v1.10.3 [37]. Salmon quantification results were then imported into R using the tximport package [38]. Differential expression analysis was conducted using the DESeq2 package [39] in R. Genes with an adjusted *P* value of less than 0.05 were considered significantly differentially expressed. The gene expression levels at 37 °C were compared to those at 25 °C. Genes with a log_2_FoldChange value lower than −0.1 were considered downregulated differentially expressed genes (DEGs), while those with a log_2_FoldChange value higher than 0.1 were considered upregulated DEGs. The volcano plot and heatmap of the DEGs were produced using the ggplot2 package [40] in R.

### Phylogenetic analysis

To construct the phylogenetic tree of *Talaromyces*, the representative genomes in this genus were downloaded from the NCBI (accession: GCA_002775195.1, GCA_023721895.2, GCF_001896365.1, GCF_001907595.1, GCA_002916415.1, GCA_004299765.1, GCA_000985935.1, GCA_022814505.1, GCA_009650675.1, GCF_009556855.1, GCA_013122295.1, GCA_000750115.2, GCA_003971505.1, GCA_031010415.1, GCA_001657655.1, GCA_001571465.2, GCF_021365285.1, GCA_019022425.1, GCA_022813215.1, GCF_013368755.1, GCF_000003125.1, GCA_037040825.1, GCA_019828575.1, GCA_020137715.1, GCA_001305275.1 and GCA_001939245.1), and the genomes were aligned by the MAFFT [41] and edited using Gblocks [42] with the options ‘-t’ conserved aa residues. The phylogenetic tree was generated using both RAxML v8.2.12 [43] and IQ-TREE v2.3.0 [44] based on 1,847 single-copy BUSCO genes. *Penicillium digitatum* (GCF_000315645.1) and *Aspergillus fumigatus* (GCF_000002655.1) were set as outgroups. The tree was edited and visualized using the online tool Interactive Tree of Life (iTOL) [45]. Whole-genome comparison of six TM genomes was conducted and visualized using AliTV [46].

### LTR identification and analysis

TransDecoder v5.7.1 [47] was used to predict ORFs in the six *T. marneffei* genomes using default parameters. The coding domains were annotated using InterProScan with the options ‘-appl pfam -dp -f TSV -goterms -iprlookup -pa -t p’. All potential retrotransposon coding sequences [containing the domain of reverse transcriptase (RT) or RNase H (RH)] were manually verified using blastp to compare their encoded protein sequences against the NCBI non-redundant protein database [48]. The LTR retrotransposon CDS encoding protein sequences longer than 500 bp were included in the subsequent phylogenetic analysis. The protein sequences were aligned and used to construct phylogenetic trees. Sequence alignment was performed by clustal W [49] and muscle [50] in mega v11 [51]. Phylogenetic trees were constructed by IQ-TREE v2.3.0 [44] and then edited by iTOL [45].

## Results

### Genome assembly and annotation of strain ATCC 18224

**Fig. 1.**
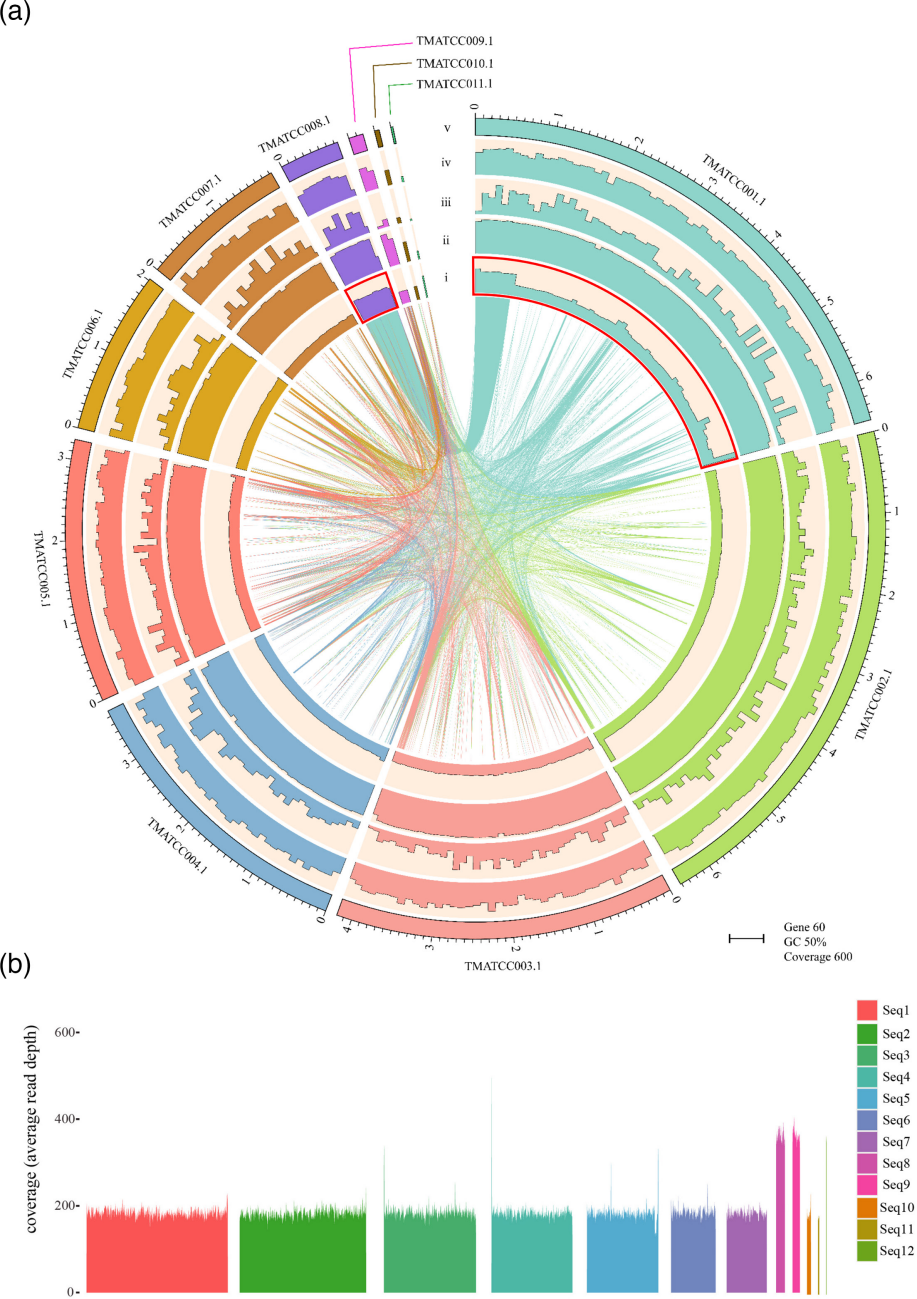
Circular plot of *T. marneffei* ATCC 18224 genome assembly and read mapping coverage of the draft genome assembly. (a) The genome assembly of *T. marneffei* ATCC 18224 was visualized in a circular plot. The outer tracks from inside include (i) genome coverage histogram, (ii) GC content histogram, (iii) simple repeat histogram and (iv) gene count histogram. The full width of the respective tracks represents 600X genome coverage, 50% GC content and 60 genes. All features were analysed using a window size of 100 kb. Genomic synteny is depicted in the centre of the plot. Two contig sequences, TMATCC001.1 and TMATCC008.1, which contain the 740-kb segmental duplication, were highlighted with red outlines, (v) length of the 11 contigs of *T. marneffei* genome assembly in megabases (Mb). (b) The read mapping coverage of 12 primary sequences (Seq1-12) in the draft genome assembly of *T. marneffei* ATCC 18224.

*T. marneffei* ATCC 18224 is the only strain of *T. marneffei* that was not isolated from a human. The genomic data of *T. marneffei* ATCC 18224 were sequenced utilizing the ONT platform, yielding 11.8 Gb data, and *de novo* assembly resulted in 11 contigs totalling 28.9 Mb (Fig. 1a). Two of the contigs (TMATCC002.1 and TMATCC005.1) contain typical telomeric repeats (CCCTAA/TTAGGG) at both ends, indicating near-complete chromosomal sequences, and six contigs (TMATCC001.1, TMATCC003.1, TMATCC004.1, TMATCC006.1, TMATCC007.1 and TMATCC009.1) contain the repeats at only one end, while no telomeric repeats were found in the other three contigs. This genome assembly was highly complete (BUSCO completeness=92.80%, duplication=3.2%). In addition to the 11 contigs, a small 4,135 bp contig was assembled with extremely high coverage (9,112X) and contains 5.8S, 18S and 28S rRNA gene sequences. Due to its small size, it is not included in our subsequent analysis. After the final genome assembly, a total of 11,304 protein-coding genes were annotated in *T. marneffei* ATCC 18224.

Compared with the previous assembly (GCA_000001985.1) that is 28.6 Mb with only 8.8X coverage (N50 : 541.5 kb), this genome assembly (GCA_046127475.1) has a substantial improvement, with a total length of 28 Mb with 240X average coverage (N50 : 4,186,281 bp). In comparison with the high-quality genome assemblies of five *T. marneffei* strains published previously (Table 1), the genome assembly sizes of the six strains ranged from 28.2 Mb (11CN-20-091) to 29.0 Mb (PM1). The N50 lengths ranged from 3,334,497 bp (PM1) to 4,186,281 bp (ATCC 18224), and the BUSCO completeness ranged from 92.8% to 93.2%. This genome assembly of *T. marneffei* ATCC 18224 is of similarly high quality to that of the other five *T. marneffei* strains.

**Table 1.** Overview of the genome assemblies of six *T. marneffei* strains

Strain	PM1	TM4	GZ8H79	11CN-03**-130**	11CN-20**-091**	ATCC 18224
Accession*	GCA_000750115.2	GCA_003971505.1	GCA_013122295.1	GCA_009650675.1	GCF_009556855.1	GCA_046127475.1
Reference	Yang *et al*. [81]			Cuomo *et al*. [82]	Cuomo *et al*. [82]	This study
Host	*Homo sapiens*	*Homo sapiens*	*Homo sapiens*	*Homo sapiens*	*Homo sapiens*	*Rhizomys sinensis*
Assembly size (bp)	29,022,615	28,307,147	28,376,234	28,216,733	28,198,338	28,872,128
BUSCO completeness†	93.00%	93.20%	93.10%	93.10%	93.10%	92.80%
BUSCO duplication	0.40%	0.30%	0.30%	0.30%	0.30%	3.20%
Assembly type	Contig	Contig	Contig	Chromosome	Contig	Contig
Number of contigs	13	8	10	9	8	11
Maximum length	6,433,212	6,574,480	6,440,398	6,376,262	6,464,005	6,490,868
N50 length (bp)	3,334,497	3,708,933	3,658,854	3,743,714	3,704,010	4,186,281

a*NCBI assembly accession.

b†BUSCO v5.4.2 with database eurotiales_odb10.

### Identification of a 740-kb segmental duplication

During the draft genome assembly of strain ATCC 18224, 12 primary sequences, Seq1-12, were initially constructed (Fig. 1b). In principle, all the assembled sequences should have consistent coverage, considering they are all single copies in the genome. Some short sequences can exhibit significantly higher coverage when they contain many repetitive sequences. For instance, the small 26 kb Seq12 showed unevenly high coverage in regions with high-repeat content (Fig. S1). However, two long assembled sequences, Seq8 (407,245 kb) and Seq9 (330,573 kb) have twice the average coverage (381X and 385X, respectively) of the first seven longest sequences, Seq1-7, of which the coverages range from 190 to 192X. Notably, in contrast to that of Seq12, the coverage of Seq8 and Seq9 was relatively consistent in most regions (Fig. 1b).

Further analysis of the raw sequencing read mapping revealed there is a connection between Seq9 and Seq8 (Fig. S2). This fragmentation may result from the high coverage of the sequences in the junction region. When aligning these primary sequences to genome assemblies of other *T. marneffei* strains, the collinearity result indicated that Seq9 and Seq8 are connected to Seq2 (Fig. S3). Our subsequent read mapping results confirmed the link between Seq8 and Seq2 (Fig. S4A). However, a 6 kb insertion, which was identified as Edge24 in the draft genome assembly by Flye assembler, was detected between Seq8 and Seq2 (Fig. S4B). To further confirm the sequence connection, PCR experiments were done using two pairs of primers designed based on Seq8, Edge24 and Seq2 (Fig. S5A), and the PCR product sizes confirmed the connection (Fig. S5B). Based on this evidence, in the final genome assembly, Seq9, Seq8, Edge24 and Seq2 were combined into a single long contig, TMATCC001.1.

However, as mentioned above, Seq9 and Seq8 exhibited twice the coverage of Seq2 in not only the draft genome assembly but also the raw read mapping (Fig. 1b). Moreover, qPCR results further support this and showed that genes from Seq9 and Seq8 have significantly lower Ct values than those from Seq2 in the genome of strain ATCC 18224 (Table S3). Therefore, we propose there is another copy of Seq8 and Seq9 in the genome of strain ATCC 18224, generated from segmental duplication. Consequently, the other copy of Seq9 and Seq8, along with Edge24, was retained and assigned to a separate 740-kb contig, TMATCC008.1 (Table S4).

To further investigate the mechanism underlying the duplication, the terminal sequences of TMATCC008.1 were extracted, translated and analysed using blastp in the NCBI database. The 3′ terminal sequences, which are also the insertion (Edge24), have a high read mapping coverage of 1,999X and match with RT, RH and integrase (INT) domains. This suggests that it is an active Ty1/*Copia* retrotransposon with LTRs. During our collinearity analysis, we found that the insertion of this retrotransposon was strain specific in strain ATCC 18224 (Fig. S3). The coverage of the 5′ terminal 2 kb sequences of TMATCC008.1 is also higher (422X) compared to the average genome coverage. These sequences match with the endonuclease domain encoded by various R1- and I-clade non-LTR retrotransposons and endonuclease-RT.

### Functional analysis of the segmental duplication

**Fig. 2.**
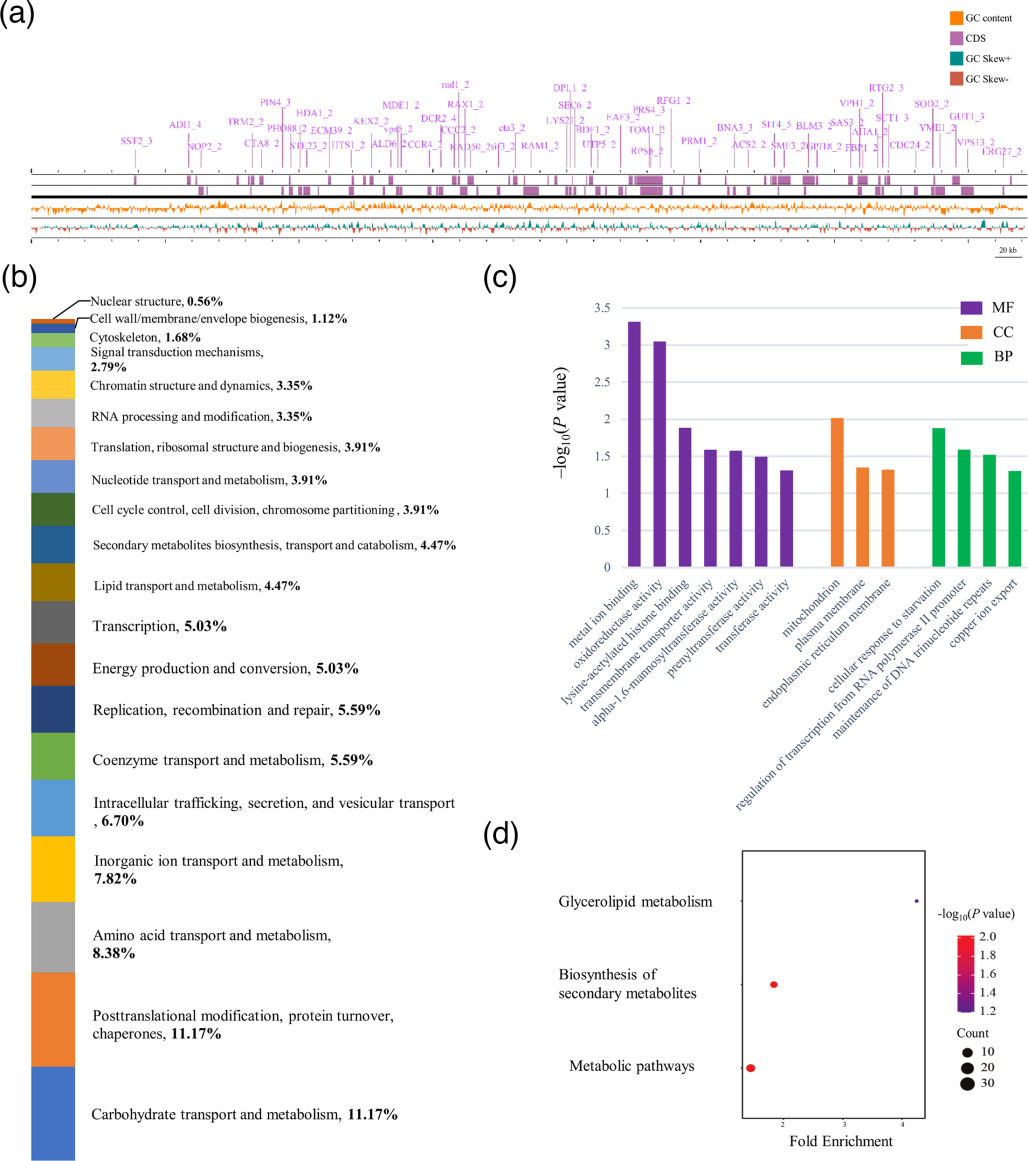
Functional annotation, cluster of orthologous groups and gene enrichment results in TMATCC008.1. (a) Linear map of TMATCC008.1 visualized by Proksee viewer. The 92 genes (Table S5) that were assigned functional annotations and gene IDs by Funannotate were highlighted. (b) COG classification of all the 299 genes annotated in TMATCC008.1. The genes were assigned and grouped into 20 COG categories. (c) KEGG pathway enrichment of the genes in TMATCC008.1. The y-axis shows the pathways, and the x-axis indicates the fold enrichment for each pathway. The bubble size represents the number of genes, while the colour bar indicates the -log_10_(*P* value). (d) GO functional annotation of the 299 protein-coding genes encoded in TMATCC008.1. The x-axis shows GO terms, and the y-axis represents the –log_10_(*P* value). Only significantly enriched GO terms (*P*<0.05) are shown here. The full table is available in Table S6.

In the duplicated sequences of TMATCC008.1, 299 protein-coding genes were annotated by MAKER annotation pipeline, and 92 genes were added with functional description by Funannotate (Fig. 2a and Table S5). To further explore the function of these genes, their annotated protein sequences were analysed by eggNOG-mapper, and a total of 261 genes were annotated in the Clusters of Orthologous Group (COG) database, distributed in 20 different COG categories (Fig. 2b). In the COG classification, the five most abundant annotated functions were carbohydrate transport and metabolism (11.17%); posttranslational modification, protein turnover and chaperones (11.17%); aa transport and metabolism (8.38%); inorganic ion transport and metabolism (7.82%); and intracellular trafficking, secretion and vesicular transport (6.70%).

In the functional enrichment analysis, the genes were significantly enriched (*P*<0.05) in 14 GO terms across 3 major categories: 4 in biological process, 3 in cellular component and 7 in molecular function (Fig. 2d and Table S6). Two molecular function GO terms were the most significantly enriched, GO:0046872 metal ion binding and GO:0016491 oxidoreductase activity (Fig. S6A). According to the KEGG enrichment analysis, 53 genes were significantly enriched (*P*<0.05) in two pathways: biosynthesis of secondary metabolites (19 genes) and metabolic pathways (34 genes) (Fig. 2c, Table S6). The gene enrichment results indicate the important roles of these genes in this segmental duplication.

**Fig. 3.**
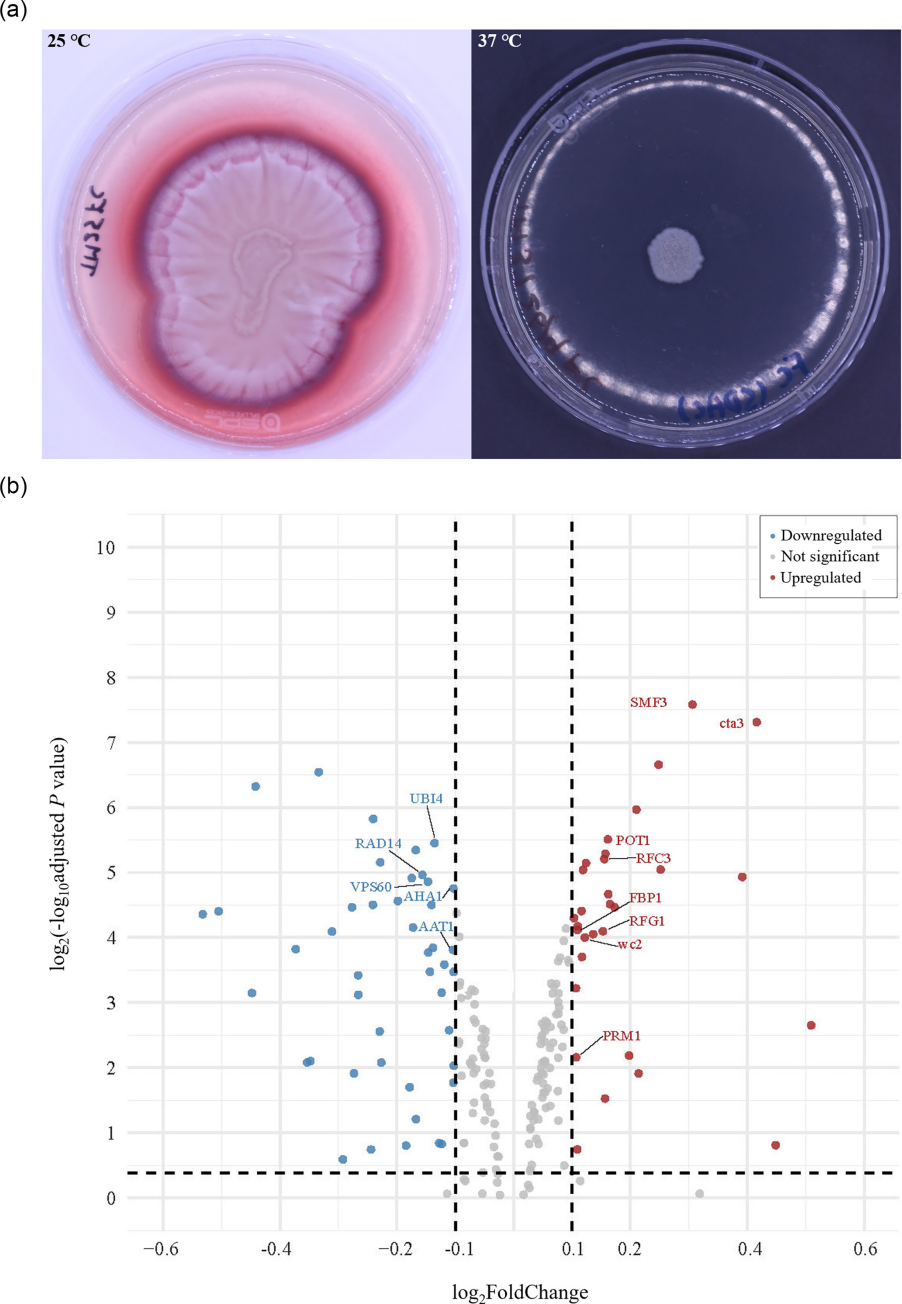
Differential expression of the genes in TMATCC008.1. (a) Morphology of *T. marneffei* ATCC 18224. The left panel shows the culture on an SDA plate (⌀ 150 mm) after 14 days of incubation at 25 °C. The filamentous colonies are yellowish with diffusing red pigment. The right panel shows the culture on an SDA plate after 7 days of incubation at 37 °C, in which the colonies are yeast like and creamy. (b) Volcano plot illustrating 75 DEGs on TMATCC008.1 when cultured at 37 °C compared to 25 °C. The x-axis represents the value of log_2_FoldChange, and the y-axis represents the log_2_ of the negative log_10_ adjusted *P* value. Among these genes, eight upregulated genes (*SMF3*, *cta3*, *POT1*, *RFC3*, *FBP1*, *RFG1*, *wc2* and *PRM1*) and five downregulated genes (*AAT1*, *AHA1*, *VPS60*, *RAD14* and *UBI4*) have been assigned functional annotations and are highlighted.

*T. marneffei* is known for its dimorphic transition, growing as filamentous at 25–30 °C and as a yeast at 37 °C [2]. To further explore the role of these duplicated genes during the dimorphic transition of *T. marneffei* (Fig. 3a), RNA-Seq data were obtained from strain ATCC 18224 cultured at 25 and 37 °C, respectively. The 299 genes in TMATCC008.1 exhibit average transcripts per million (TPM) values of 182.30 at 25 °C and 146.51 at 37 °C, significantly higher than the genome-wide average of 91.23. In contrast, the 267 genes in the collinear region of strain PM1 show no significant difference in average TPM values, with 81.28 at 25 °C and 81.45 at 37 °C, compared to the whole-genome average of 94.64. This indicates that the duplicated genes in strain ATCC 18224 increased expression level at both temperatures and tend to be regulated by dimorphic transition, which were not observed in the strain PM1. This reflects the dosage effect where the duplication of gene sequences results in higher expression levels. Using a cutoff of |log2(FoldChange)|≥0.1 and *P* value<0.05, we identified 75 DEGs from the 299 annotated genes in the duplication: 45 were significantly downregulated and 30 were significantly upregulated (Fig. 3b). In the gene enrichment analysis, eight GO terms were significantly enriched, including the molecular function term GO:0046872 metal ion binding (Fig. S6). Among these, five downregulated genes (*UBI4*, *RAD14*, *VPS60*, *AHA1* and *AAT1*) and eight upregulated genes (*SMF3*, *cta3*, *POT1*, *RFC3*, *FBP1*, *RFG1*, *wc2* and *PRM1*) were functionally annotated. A similar DEG analysis in the collinear region of the duplication was performed on strain PM1, identifying eight functionally annotated genes (*cta3*, *HDA1*, *SST2*, *MNN11*, *YPD1*, *PRM1*, *GUT1* and *HTZ1*) that were downregulated and five (*YME1*, *SMF3*, *UTP5*, *DYS1* and *NOP2*) that were upregulated. Only three DEGs overlapped between the two strains. Among these, the metal ion transporter gene *SMF3* was upregulated in both strains, while *cta3* and *PRM1* showed opposite differential expressions between the strains.

### Comparative genomic analysis of six *T. marneffei* strains

To further explore the genomic structural variation, high-quality genome assemblies of six *T. marneffei* strains were comparatively analysed (Table 1). Regarding the segmental duplication, we have downloaded the raw sequencing data for the other four strains [excluding strain TM4 without raw data, and the old version of ATCC 18224 genome (GCA_000001985.1) has no available raw sequencing data] and confirmed that there is no such duplication by comparing the read mapping coverages. Given the absence of the LTR retrotransposon insertion, we found no evidence that strain TM4 contains a similar segmental duplication. Therefore, we propose that the 740-kb segmental duplication is specific to strain ATCC 18224.

**Fig. 4.**
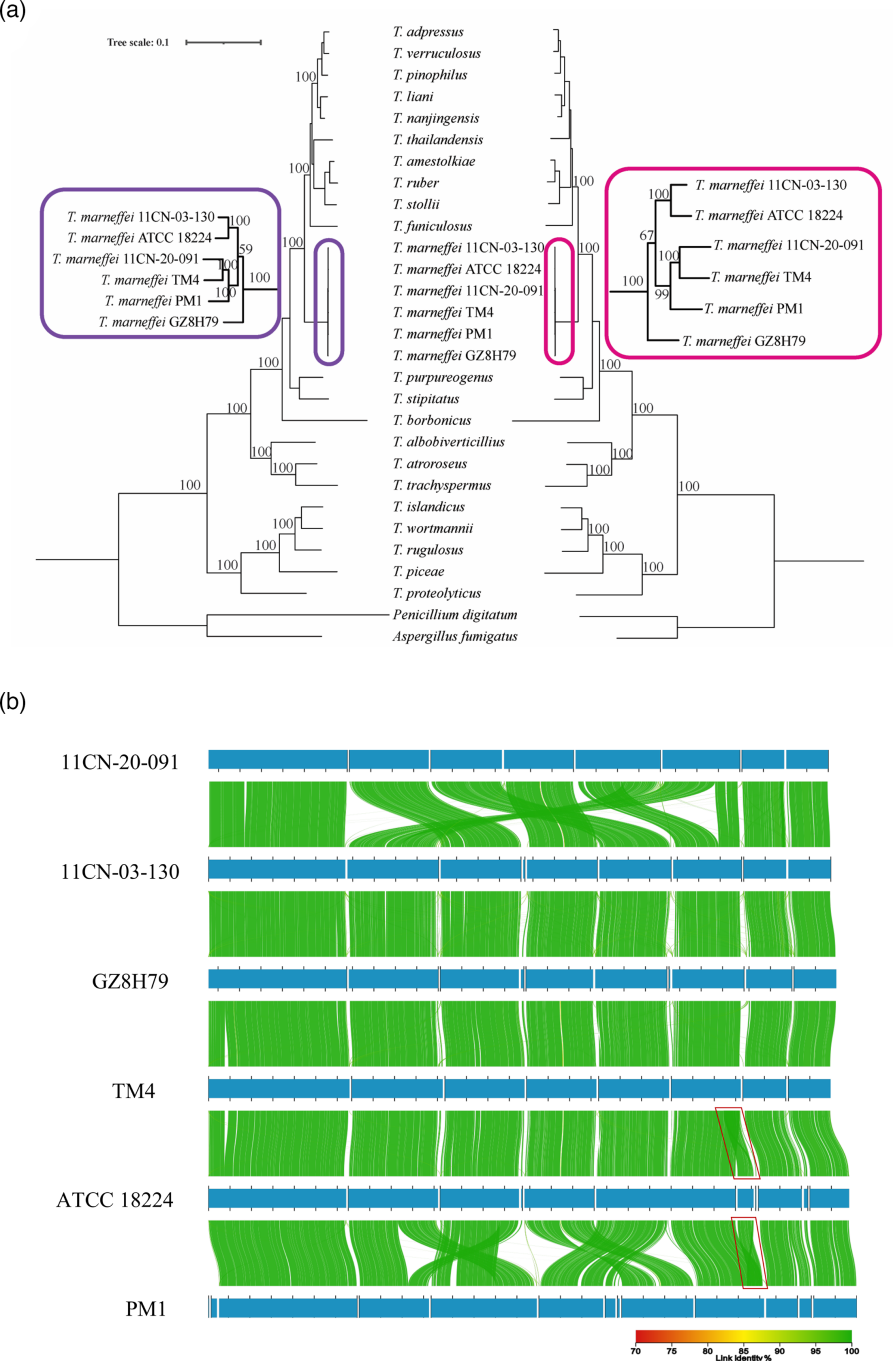
Phylogenomic analysis of *Talaromyces* species and whole-genome alignment of six *T. marneffei* strains. (a) Phylogenomic trees of 27 *Talaromyces* species were constructed using RAxML (left) and IQ-TREE (right) with 1,000 bootstrap replicates, based on the protein sequence alignment of 1,847 overlapping single-copy BUSCO genes. The clade containing six *T. marneffei* strains is highlighted in two zoomed-in windows. *P. digitatum* and *A. fumigatus* were used as outgroups. (b) Whole-genome alignment of six *T. marneffei* genomes visualized using AliTV. The alignment was filtered by 70% identity and an over 15 kb link length. The synteny connections labelled homologous genomic regions among the genome assemblies of six *T. marneffei* strains based on their nt similarities. The contig TMATCC008.1, generated by the segmental duplication in strain ATCC 18224, is highlighted by black triangles.

Phylogenetic trees were constructed for 29 genome assemblies (Fig. 4a). Two topology-consistent phylogenetic trees were generated using RAxML and IQ-TREE 2, based on 667,803 conserved aa residues from 1,847 overlapping single-copy BUSCO protein sequences (eurotiales_odb10 database). *T. marneffei* was positioned as an outgroup relative to the other ten non-pathogenic *Talaromyces* species. Within the genus, it is the only species documented as a well-known human pathogen. *Talaromyces stipitatus*, the closest known relative of *T. marneffei* [52], and *Talaromyces purpurogenus*, a species commonly found in marine environments [53], were clustered together and positioned outside the six *T. marneffei* strains in the phylogenetic tree. In the phylogenetic trees, the six *T. marneffei* strains formed a distinct cluster, separate from other *Talaromyces* species. The exceedingly short branches within this cluster were indicative of exceptionally high protein sequence identities (over 99.9%) among the six strains. Within this cluster, strain GZ8H79 was positioned outside, while strains 11CN-03–130 and ATCC 18224 formed one sub-cluster, and the remaining three strains formed another sub-cluster.

However, in the whole-genome alignment of the six *T. marneffei* strains (Fig. 4b), we observed consistent collinearity among strains 11CN-03-130, GZ8H79, TM4 and ATCC 18224, which contrasts with the phylogenetic tree findings (Fig. 4a). The alignment revealed various large-scale translocation and inversion events in strains PM1 and 11CN-20-091, compared to the other four conserved strains. Additionally, when examining smaller genomic segments (1–15 kb) among the assemblies (Fig. S7), we identified numerous translocated homologous sequences, primarily resulting from the active expansion of transposable elements. Taken together with the strain-specific insertion of the Ty1/*Copia* retrotransposon in ATCC 18224, these results indicate significant variations in transposable elements among the *T. marneffei strains*.

### Variable Ty1/*Copia* retrotransposons in *T. marneffei*

In the *de novo* identification of repetitive elements, the repeat content of the six *T. marneffei* strains varied from 5.00% in strain 11CN-20-091 to 5.82 % in strain GZ8H79. The content of LTR retrotransposons ranged from 0.85% in strain PM1 to 1.46% in strain ATCC 18224. On the other hand, long interspersed nuclear elements (LINEs) ranged from 0.59% in strain ATCC 18224 to 1.21% in strain 11CN-20-091 (Table S8). In fungal genomes, LTR retrotransposons are the most abundant and well-characterized transposable elements, primarily belonging to two main superfamilies: Ty1/*Copia* and Ty3/*Gypsy* [54]. For this reason, we have focused on LTR retrotransposons to explore their variations among the *T. marneffei* strains.

Through further analyses, we discovered that many LTR elements found in the RepeatMasker output were incorrect. Specifically, most of them were incomplete in the coding region, and many short, fragmented sequences were included. Other commonly used tools for LTR identification, such as LTR_FINDER, were only able to identify a few LTR elements with low accuracy. Therefore, a more straightforward method to analyse the LTR elements was employed. Given that the polyproteins of LTR retrotransposons have no introns, TransDecoder was used to extract ORFs from the genome sequences of *T. marneffei*. Functional annotation using eggNOG-mapper was then performed to search for key domains of LTR elements, including protease (PR), INT, RH and RT, and finally manually validated in the NCBI blastp online.

A total of 301 LTR ORFs were identified in the 6 *T. marneffei* genomes (Table S9). According to the previous studies [54], a complete ORF of LTR polyproteins encodes PR, INT, RH and RT. However, no PR domain was identified in these ORFs, and many ORFs encode only one or two domains. In the Ty1/*Copia* family, 120 ORFs encode RH, RT and INT; 62 encode RH and RT; and 32 encode only RT. In the Ty3/*Gypsy* family, 8 ORFs encode RH, RT and INT; 42 encode RH and RT; and 37 encode only RT. More LTR ORFs were found in the Ty1/*Copia* family (214) than in the Ty3/*Gypsy* family (87). In addition, higher variation in ORF counts among the six *T. marneffei* strains was observed within the Ty1/*Copia* family, prompting further investigation.

**Fig. 5.**
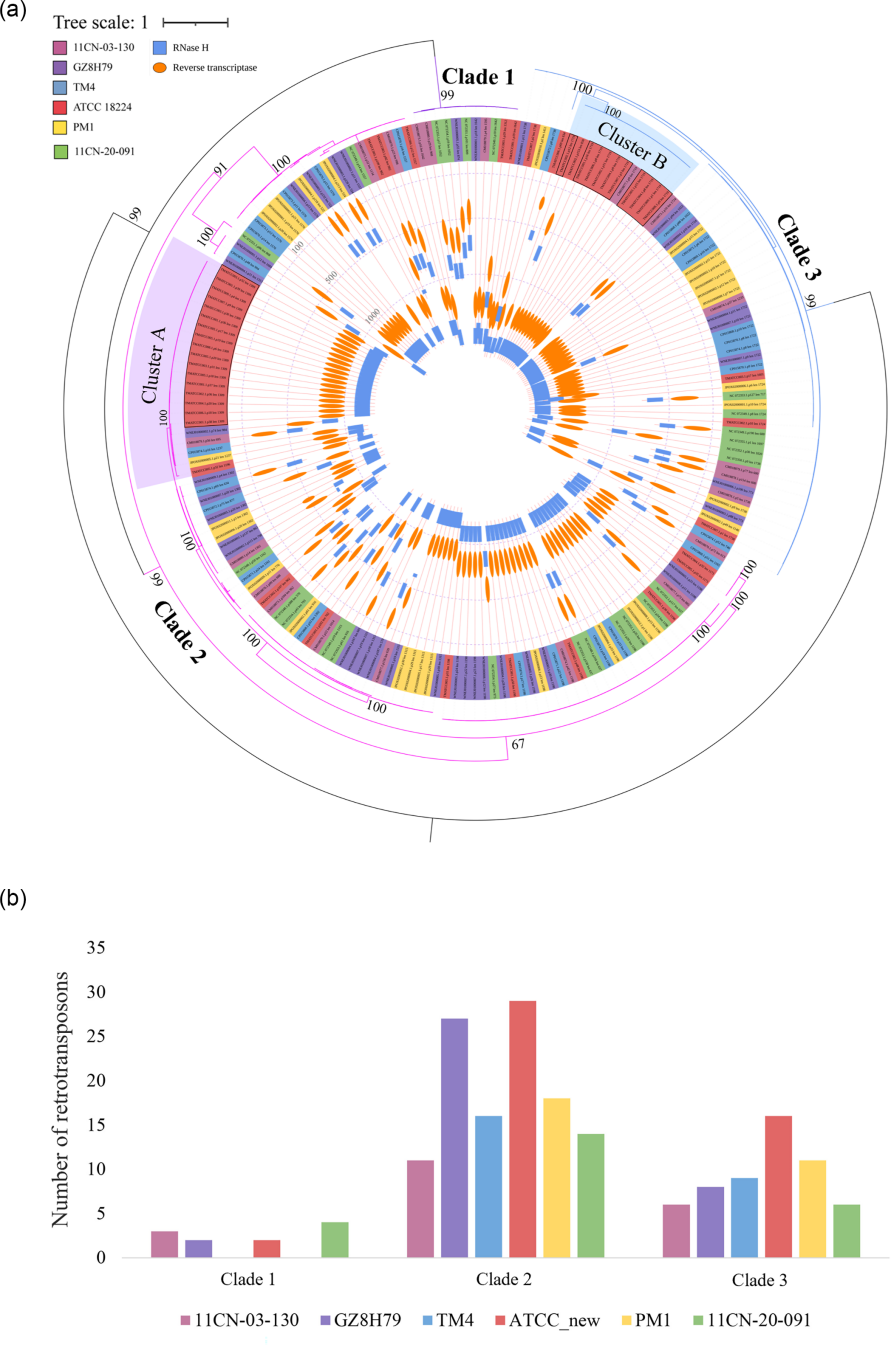
Phylogenetic analysis of Ty1/*Copia* retrotransposons identified in six *T. marneffei* strains. (a) Phylogenetic tree of Ty1/*Copia* retrotransposons in six *T. marneffei* strains. This tree was constructed using IQ-TREE based on the protein sequence alignment of concatenated ribonuclease H (RH) and RT domains, with 1,000 bootstrap replicates. The entire phylogenetic tree is divided into three clades (1–3). Two clusters (A and B) containing multiple copies in strain ATCC 18224 are highlighted. Within these two clusters, 17 and 10 strain-specific LTRs of strain ATCC 18224 share identical sequences and are labelled with black outlines. Inside the tree, rectangular and elliptical modules represent RH and RT domains, respectively. The positions in the inner circle represent the locations of RT and RH within the polyprotein sequences. The three dashed circular lines indicate positions of 100, 500 and 1,000 aa from the outside to the inside. (b) The number (y-axis) of retrotransposons in each clade for the six strains. Clade 2 is the largest and most variable, Clade 3 is the second largest and Clade 1 is the smallest and contains none from strains TM4 and PM1.

Based on their encoded protein sequences, these LTR ORFs were further analysed using phylogenetic trees. The phylogenetic tree of the Ty1/*Copia* family revealed three major clades. According to the results from the phylogenetic tree of concatenated RH and RT (Fig. 5a), Clade 1 is the smallest and highly conserved, containing only 11 LTRs, with no LTRs found in strains TM4 and PM1 (Fig. 5b). Clade 2 is the largest and most variable in length. Within this clade, Cluster A was particularly notable. In Cluster A, a significant expansion was observed specifically in strain ATCC 18224, resulting in 17 strain-specific LTRs (highlighted with a black outline) that encode an identical long polyprotein of 1,309 aa. Interestingly, this highly expanded LTR in ATCC 18224 shares an identical sequence with the previously mentioned Edge24. Compared to Clade 2, Clade 3 is smaller and less variable. Within Clade 2, Cluster B was highlighted for its recent expansion in strain ATCC 18224, resulting in 10 identical LTR ORFs (highlighted with a black outline) that encode a long polyprotein of 1,719 aa. This long polyprotein ORF of 1719 aa has a homologue with an identical sequence in strain GZ8H79. Transcriptome data analysis of strains ATCC 18224 and PM1 revealed that most of their Ty1/*Copia* LTRs are actively expressed. Notably, the expression level of Cluster A in ATCC 18224 was relatively low at 25 °C (average read number: 675) but absent at 37 °C (Table S10). A similar phylogenetic tree was constructed for the LTR ORFs of the Ty3/Gypsy family (Fig. S8), identifying a recently expanding cluster of LTRs in strain 11CN-20-091.

## Discussion

In this study, a high-quality genome of *T. marneffei* ATCC 18224 was assembled in 28.9 Mb and 11 contigs, based on third-generation ONT sequencing reads. The high continuity and completeness supported the high quality of this genome assembly. This genome assembly, based on ONT sequencing, represents a significant improvement over the initial assembly performed using Sanger sequencing with 8.8X coverage in 2008 [55]. In our subsequent analysis of this genome assembly, we identified a 740-kb segmental duplication event specific to strain ATCC 18224, which contributed 2.9% BUSCO duplication rate (3.20%) in the final genome assembly. According to our analysis of the terminals of the duplicated sequence of TMATCC008.1, we propose that this segmental duplication was mediated by the insertion of a Ty1/*Copia* LTR retrotransposon, which was named Edge24 in the draft genome assembly. In the yeast *S. cerevisiae*, Ty elements or retrotransposons have been reported to cause chromosomal rearrangements [56,58] and segmental duplications [59,60] via homologous recombination. However, such a large segmental duplication of 740 kb is rarely reported in fungi [60].

This 740-kb segmental duplication represents a significant and strain-specific genomic structural variation in *T. marneffei* ATCC 18224. Within this duplicated region, a total of 299 protein-coding genes were annotated. Of these, 92 genes were functionally annotated by Funannotate (Table S5), and 261 genes were assigned to COG categories (Fig. 2a). Differential gene expression analysis further identified 75 genes that can be possibly related to the dimorphic transition of *T. marneffei*, including 13 genes that have functional annotation. The enrichment analysis of both the duplicated genes and the DEGs revealed a significant number of genes related to metal ion binding (Figs S6 and 7). This finding is particularly interesting because metal ion binding and transport are critically important in fungal stress response and adaptation [61]. Thus, this segmental duplication may represent an adaptive advantage for strain ATCC 18224. However, the distinct DEGs between strains ATCC 18224 and PM1 highlight the intraspecific variations within *T. marneffei*. Although further functional analysis of these segmental duplications and associated genes would be valuable, it falls outside the primary focus of this study, which is centred on genomic structural variation.

To further investigate the genomic structural variation of *T. marneffei*, we performed a comparative genomic analysis on the genome sequences, where the structural rearrangement landscape between the six strains of *T. marneffei* was first revealed. The multi-locus phylogenetic tree, based on 1,847 overlapping single-copy BUSCO protein sequences, shows a topology that is generally consistent with previous studies [62,65]. Eight sections of *Talaromyces* have been established, *Bacillispori*, *Helici*, *Islandici*, *Purpurei*, *Subinflati*, *Talaromyces*, *Tenues* and *Trachyspermi* [65,66]. Genome sampling for this study covered *sect. Talaromyces* (e.g. *T. marneffei*), *sect. Helici* (*Talaromyces borbonicus*), *sect. Trachyspermi* (*Talaromyces albobiverticillius*, *Talaromyces atroroseus* and *Talaromyces trachyspermus*), *sect. Islandici* (*Talaromyces islandicus*, *Talaromyces wortmannii*, *Talaromyces rugulosus* and *Talaromyces piceae*) and *sect. Bacilispori* (*Talaromyces proteolyticus*). These sections represent monophyletic groups in our phylogenetic tree, demonstrating clear and distinct evolutionary lineages. Compared to phylogenetic trees constructed using limited genes or sequences, such as the mitochondrial genome or internal transcribed spacer (ITS) region [67,68], this phylogenetic tree, which involves 1,847 overlapping protein sequences, is more robust and convincing. This phylogenetic tree divides the six *T. marneffei* strains into at least three clusters: strain GZ8H79 is located outside, strains 11CN-03-130 and ATCC 18224 comprise one sub-cluster and the remaining three strains constitute a separate sub-cluster. Notably, the ITS regions and 18S rRNA sequences are identical across all six *T. marneffei* strains and cannot differentiate them. However, in the whole-genome alignment, we observed an inconsistent grouping of the six strains. Large-scale genome rearrangements were only observed in strains PM1 and 11CN-20-091, while the other four strains shared conserved synteny. In addition to the large rearrangements, various small rearrangements and active transposable elements were observed mostly at the terminal regions in the whole-genome alignments.

This inconsistency between the sequence alignment-based phylogenetic tree and whole-genome rearrangement raises the necessity for future phylogenomic studies. We believe that rearrangements should be taken into consideration in phylogenetic and comparative genomic studies of *T. marneffei*, especially considering it has no sexual stage discovered yet [69]. In this case of no obvious sexual stage, chromosomal rearrangements are important for their diversity and adaptation [70,71]. For instance, genome rearrangements in *Verticillium dahliae*, a fungal plant pathogen, generated highly dynamic lineage-specific regions that enrich planta-expressed genes, which contributed to host infection [70,72].

It has been frequently reported that fungal genome recombination can be mediated by LTR retrotransposon and contribute to genome plasticity and adaptation [73]. Given the variations in transposable elements observed among *T. marneffei* strains in our study, we specifically examined the variations of LTR retrotransposons, focusing on the Ty1/*Copia* family. Retrotransposons are transposable elements that exhibit significant functions in fungal genomes. According to previous studies, LTR retrotransposons can take part in the adaption to stress under a severe adenine deficiency situation by coactivating genes adjacent to Ty1 sequences and regulating the initiation of transcription at alternative sites in *S. cerevisiae*, or promoting host infection of plant pathogens *Botrytis cinerea* [74,75], also providing a source of phenotypic and genotypic plasticity by forming extrachromosomal circular DNAs in the rice blast pathogen *Magnaporthe oryzae* [76]. However, understanding of LTRs in human fungal pathogens is still very lacking.

Due to the high variability of the PR domain and the limited sequences in reference databases, no PR domain was identified. Additionally, the incomplete pol-encoded protein domains (INT, RH and RT) observed in many elements, often lacking one or more core components, may reflect their degraded or non-functional state. These highly active Ty1/*Copia* retrotransposons contribute to the genetic variation and diversity of *T. marneffei*, potentially affecting their pathogenicity evolution and even host adaptation. In addition, the multiple copies generated by the active duplication of these retrotransposons cause significant challenges to our genome assembly. Next-generation sequencing (NGS) short reads that cannot span these long retrotransposons of over 6 kb could not achieve high-quality and highly continuity genome assembly [77]. During the identification of retrotransposon, we did not find any good pipelines or tools that were suitable for *T. marneffei*; one possible reason is that they were not developed for fungi. Although our customized identification method cannot fully resolve complete structures, such as LTR repeats, and the lack of homology makes it difficult to identify functional domains, the presence of multiple copies and evidence of active transcription suggest their potential functionality and activity. Similarly, in the ectomycorrhizal fungus *Laccaria bicolor*, the expansion of LTR retrotransposons has been reported to contribute to species divergence [78]. Therefore, we believe a more comprehensive investigation of transposable elements, especially LTR retrotransposons, needs attention not only in the identification pipeline but also in the comparative analysis among *T. marneffei* strains.

However, this study has several major limitations. First, the genome assembly is incomplete, as not all contigs have telomeric repeats at both ends. Consequently, we cannot confirm the location of the additional copy of the 740-kb segmental duplicated sequence or determine whether it is independent, due to the incompleteness of the genome assembly. Second, there is a lack of effective tools for identifying the transposable elements of *T. marneffei*, which hinders our understanding of the active retrotransposons. Lastly, a limited number of high-quality genomes of *T. marneffei* strains have prevented us from fully investigating the whole-genome comparison, classification and evolution of *T. marneffei* strains. In contrast, a series of high-quality comparative genomic studies on *Candida auris* have been conducted to explore their genome variation and identify clade-specific rearrangements [79,80], which raises the importance of a more comprehensive comparative genomics study of *T. marneffei* in the future.

## Conclusions

In this study, extensive genomic structural variation in *T. marneffei* was revealed through a high-quality genome assembly of strain ATCC 18224 and a comparative genomic analysis of six strains. The 740-kb segmental duplication in strain ATCC 18224 constitutes a notable genomic structural variation in the genome, resulting in 299 duplicated protein-coding genes that significantly enriched molecular functions such as metal ion binding. In the comparative analysis, we identified large-scale chromosomal rearrangement events in two *T. marneffei* strains, PM1 and 11CN-20-091, which contradict the multi-locus phylogenetic trees based on protein sequence variations. Further investigation of the Ty1/*Copia* retrotransposon uncovered substantial genetic variations among *T. marneffei* strains, including a series of recent expansion events. These extensive genomic structural variations in *T. marneffei* will enhance our understanding and pave the way for improved genotyping, classification and genome surveillance of this important pathogenic fungus.

## Supplementary material

10.1099/mgen.0.001400 Uncited Supplementary Material 1.

## Abbreviations

ATCC: American Type Culture Collection
BUSCO: Benchmarking Universal Single-Copy Orthologs
CDS: coding DNA sequences
COG: Clusters of Orthologous Group
DEGs: differentially expressed genes
GO: Gene Ontology
iTOL: Interactive Tree of Life
ITS: internal transcribed spacer
LTR: long terminal repeat
ONT: Oxford Nanopore Technologies
PR: protease
RH: RNase H
RT: reverse transcriptase
SDA: Sabouraud dextrose agar
SDB: Sabouraud dextrose broth
TPM: transcripts per million
