# Supplementary material for: High-quality genome assembly and comparative analysis reveal extensive genomic variation in Talaromyces marneffei
Source: Microb Genom. 2025 Apr 28;11(4):001400. doi: 10.1099/mgen.0.001400 (PMC12037069; doi:10.1099/mgen.0.001400)
Supplement: Uncited Supplementary Material 1. [file mgen-11-01400-s001.pdf]

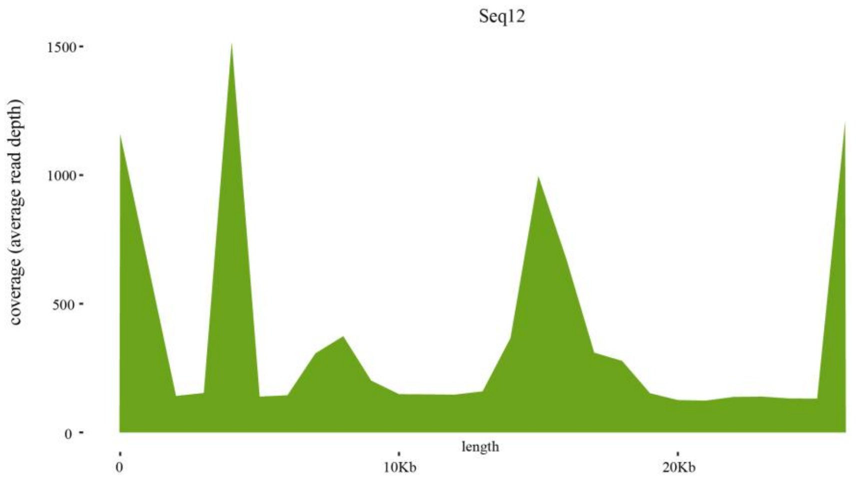

Figure S1. Read mapping coverage of Seq12 in the draft genome assembly

The coverage is distributed unevenly in the Seq12.

A

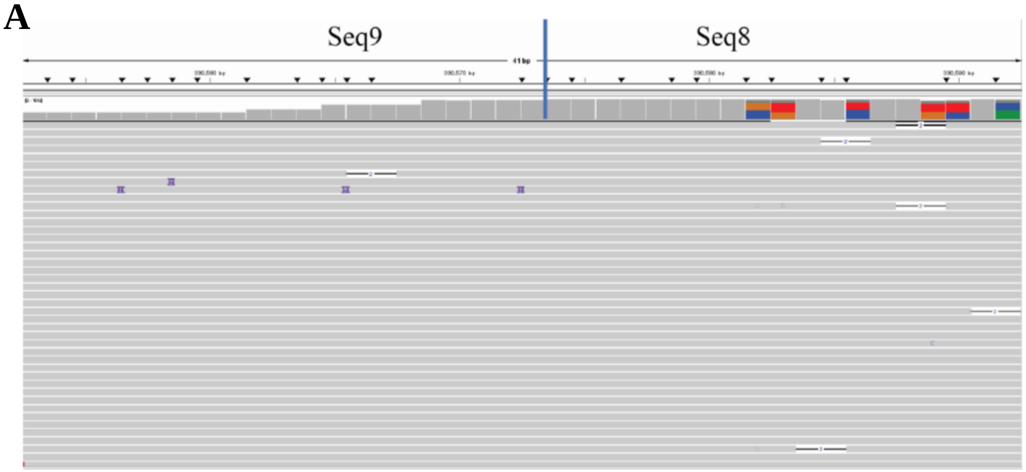

B

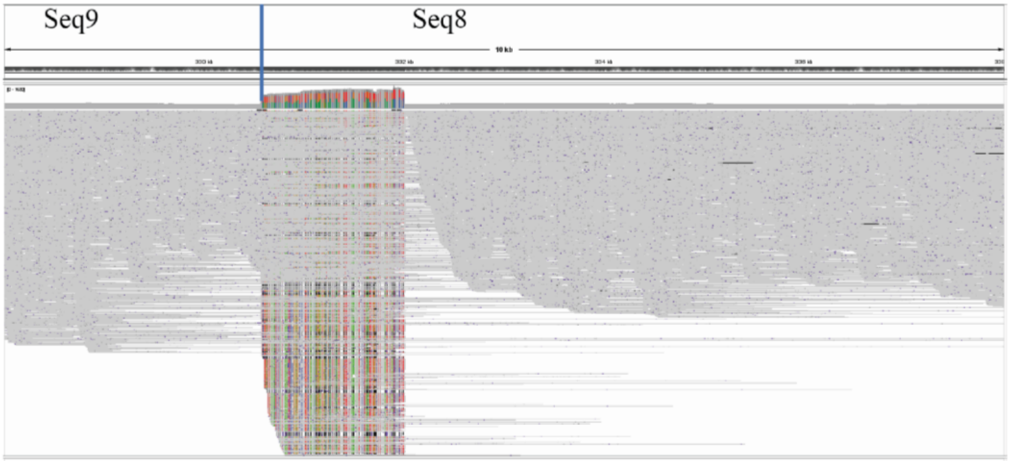

Figure S2. The junction between Seq9 and Seq8

Raw ONT sequencing reads were mapped to the junction between Seq9 and Seq8. The upper panel displays a 41 bp window, showing detailed alignment of reads to the reference sequence. Gray boxes represent aligned portions of the reads, while gray lines between boxes indicate gaps or spanned regions where the reads do not directly align to the reference genome. Numbers on the gray lines denote the number of reads supporting the spanned region. Purple boxes highlight structural variations, such as insertions or deletions, with the numbers inside representing the size of the variation in base pairs. The lower panel shows a 10 kb window, providing a broader view of read coverage across the junction, where colored blocks represent mismatches or variations, and gray lines represent aligned reads. This mapping result indicates that Seq9 and Seq8 are connected.

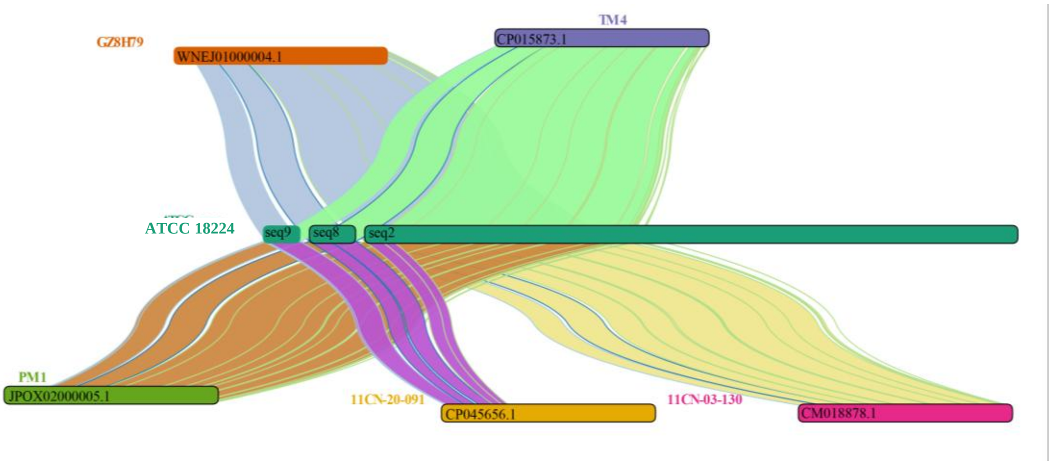

Figure S3. Collinearity analysis of Seq9, Seq8 and Seq2 compared to other five *T. marneffei* strains

Collinearity analysis conducted by GenomeSyn revealed that the homologous sequences of Seq9, Seq8, and Seq2 in five other *T. marneffei* strains are connected in the same order.

A

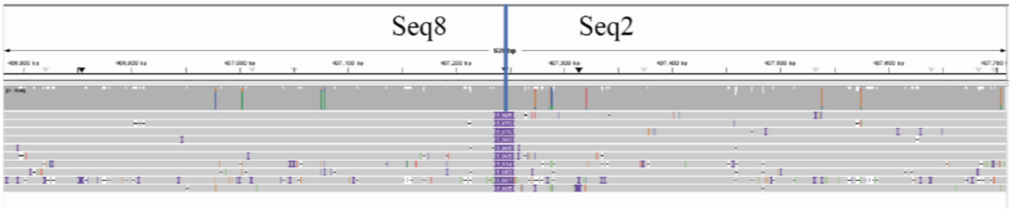

B

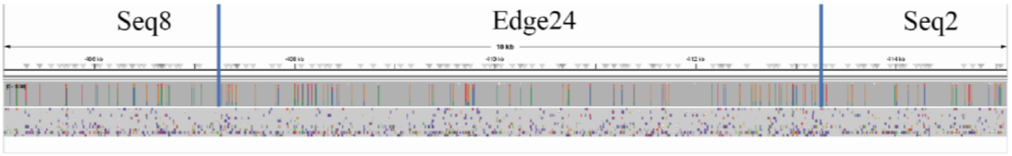

Figure S4. Raw sequencing read mapping of Seq8, Edge24 and Seq2

(A) The raw ONT read mapping at the junction of Seq8 and Seq2 shows a 6-kb insertion between Seq8 and Seq2. **(B)** After manually adding Edge24 between Seq8 and Seq2, the mapping results of raw ONT reads show that Seq8, Edge24, and Seq2 are connected. Colored blocks represent mismatches or variations, and gray lines represent aligned reads.

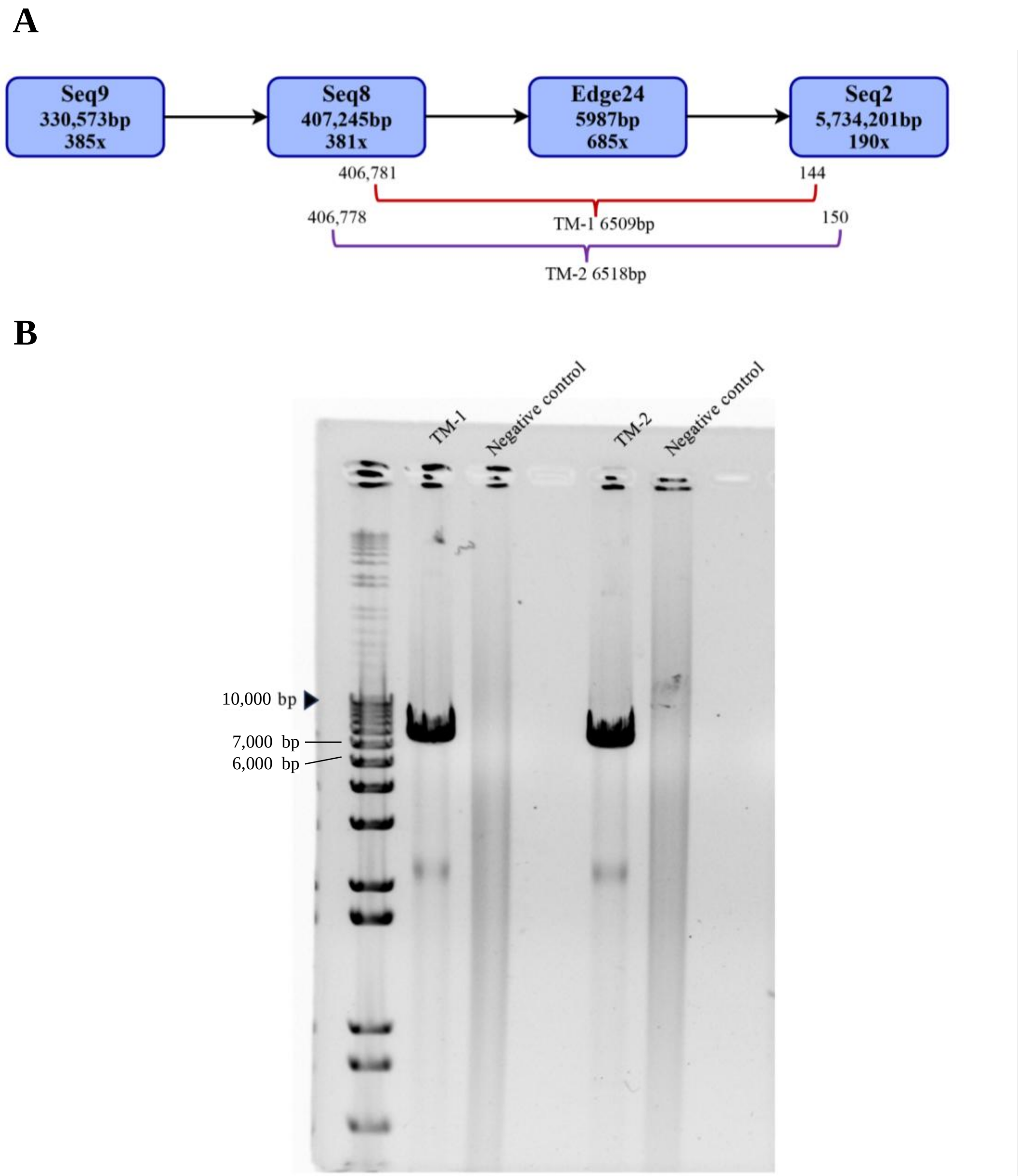

Figure S5. PCR confirmed the insertion of Edge24 between Seq8 and Seq2

(A) A simplified graph illustrates the draft genome assembly of *T. marneffei* ATCC 18224. Two pairs of PCR primers were designed on Seq8 and Seq2 to span Edge24. **(B)** Gel electrophoresis of the PCR products shows that the product sizes are approximately 6 kb, supporting the insertion of Edge24 between Seq8 and Seq2. An equal volume of distilled water was used as the negative control in place of DNA.

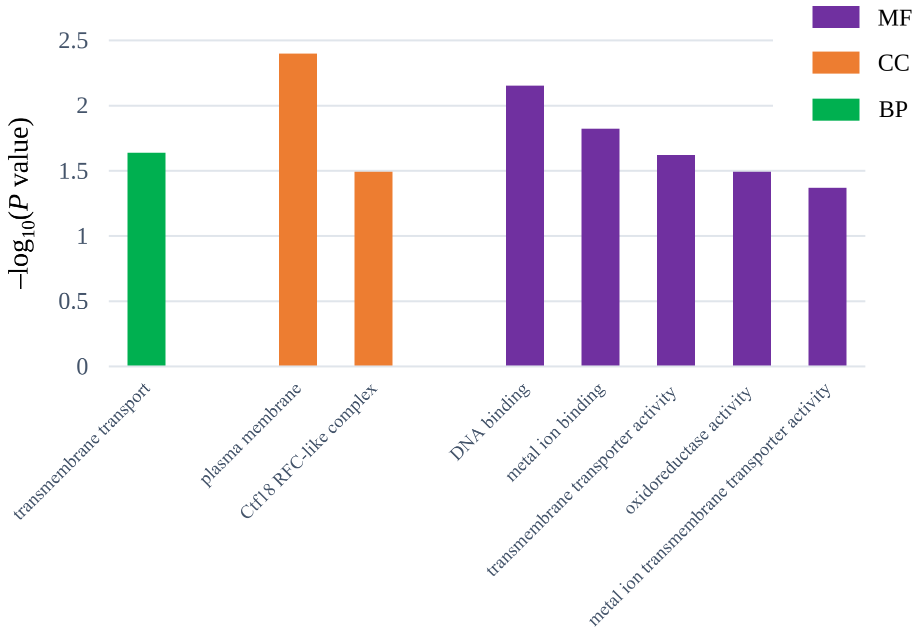

Figure S6. Gene enrichment of the 75 DEGs in TMATCC008.1

GO functional annotation of the 75 DEGs in TMATCC008.1 when *T. marneffei* is cultured at 37°C versus 25°C. The x-axis shows GO terms, and the y-axis represents the $-\log_{10}(P$ value). Only significantly enriched GO terms ($P < 0.05$) are shown here. The full table is available in Table S6.

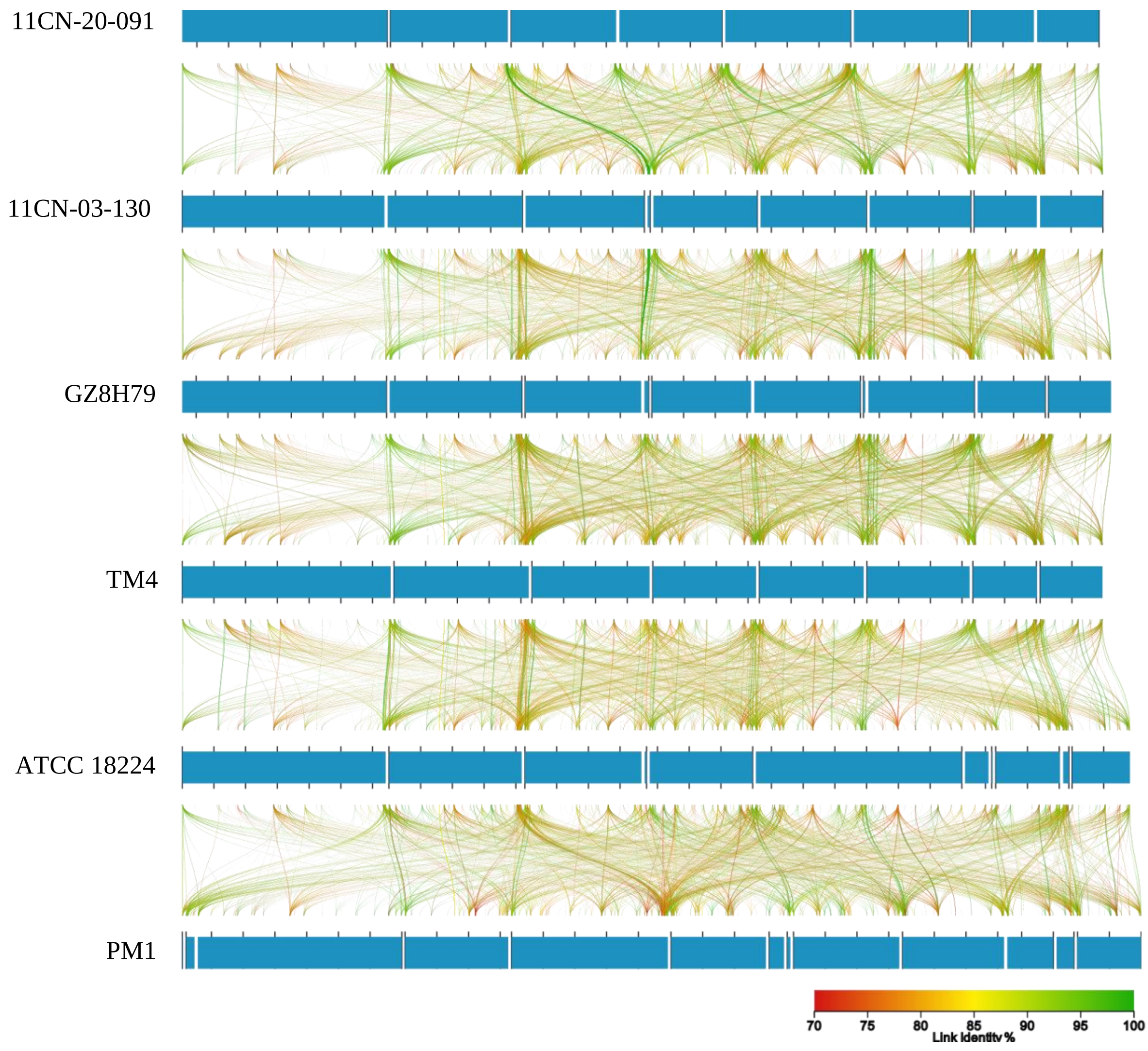

Figure S7. Whole genome alignment of six *T. marneffei* genomes

The alignment was filtered by 70% identity and a link length of 1-15 kb. This length range covers most transposable elements.

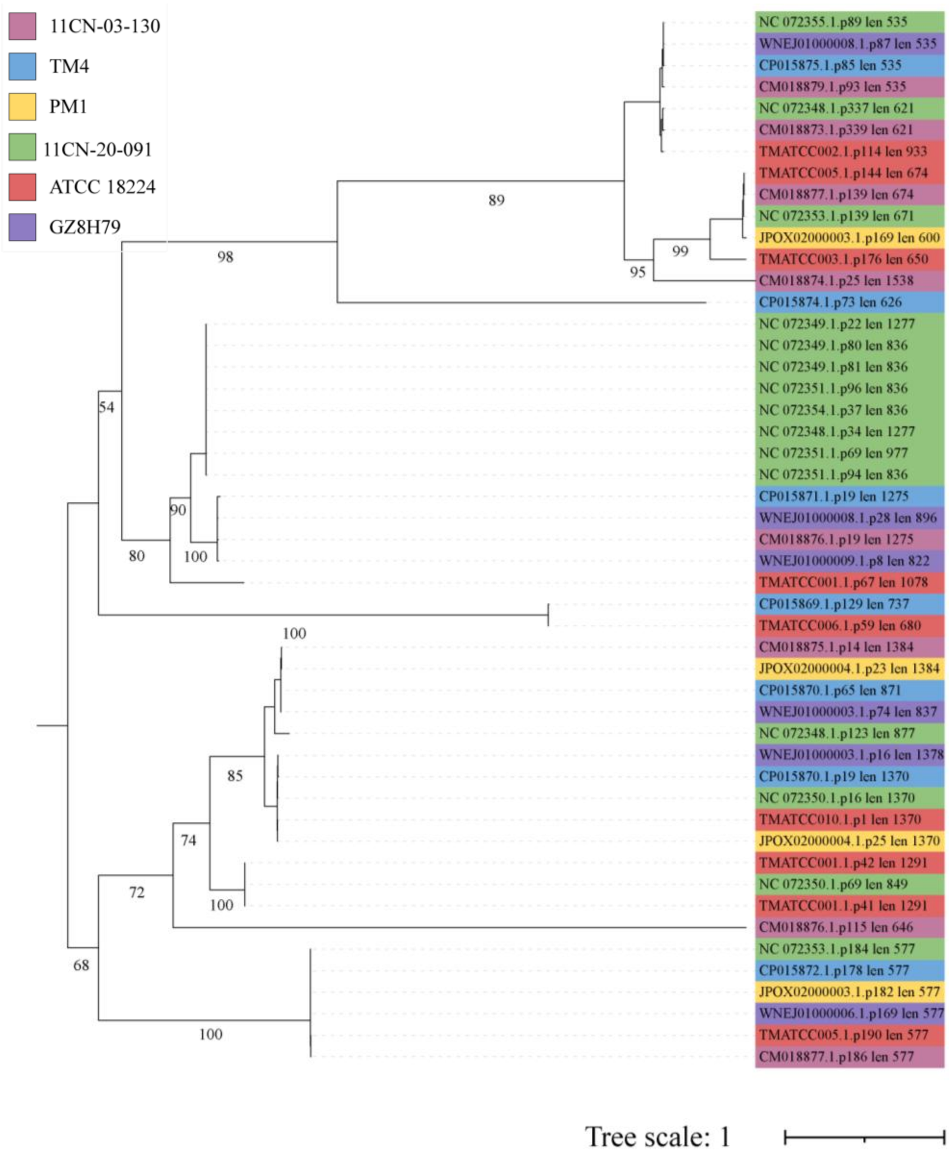

Figure S8. Phylogenetic analysis of Ty3/*Gypsy* retrotransposons

This tree was constructed using IQ-TREE based on the protein sequence alignment of concatenated ribonuclease H (RH) and reverse transcriptase (RT) domains, with 1,000 bootstrap replicates.

Table S1. Quality assessment of RNA sequencing data

Sample	Accession	Number of reads	Q20 (%)	Q30 (%)	GC (%)	Mapping rate (%)
25_1	SRR31006959	21,986,540 *2	97.41	93.18	49.71	90.39
			97.3	92.72	49.8	
25_2	SRR31006958	20,341,487 *2	97.17	92.89	49.91	87.71
			97.02	92.2	49.73	
37_1	SRR31006957	24,903,429 *2	97.3	93.03	49.73	89.69
			97.28	92.68	49.72	
37_2	SRR31006956	21,778,822 *2	97.42	93.2	49.76	89.83
			97.17	92.43	49.68	

Table S2. Primers used in this study

Primer	Type	Primer pair(5'-3')	Product length (bp)	Location	GC%
P1	qPCR forward	AAGGCAAAGACTCAGTGCGA	216	TMATCC008.1	50
	qPCR reverse	ATTACAGCGTCCCAAGTAGC			50
P2	qPCR forward	GCCTTTGCGCTGTGAGATTT	92	TMATCC008.1	50
	qPCR reverse	ATGGCTGGCGGTAAAGGAAA			50
P3	qPCR forward	TCAATGCTGACACCATGACATC	142	TMATCC008.1	45.45
	qPCR reverse	ACACTCACACTGCCAAAACC			50
P4	qPCR forward	GCTCCTCGTAAATGCTGCTC	202	TMATCC001.1	55
	qPCR reverse	GTCATCTGCGAAAAGGTCGAG			52.38
P5	qPCR forward	GCCAGGAGGACCGATCAAG	161	TMATCC001.1	63.16
	qPCR reverse	CTCCCATCACGCAAGAGACC			60
P6	PCR forward	TGGAATGGGCAAGGTAAATGC	6509	TMATCC001.1 TMATCC008.1	47.62
	PCR reverse	ATCCCATGATTCGACGCTCCT			52.38
P7	PCR forward	GCCTGGAATGGGCAAGGTAAAT	6518	TMATCC001.1 TMATCC008.1	50.00
	PCR reverse	ATATCAATCCCATGATTCGACGCT			41.67

Table S3. qPCR results of *T. marneffei* ATCC 18224

Primer name	Ct value	Tm	GC proportion(%)	Product length
P1	14.06	84.53	50/50	216
P1	14.2	84.53	50/50	216
P2	14.94	85.57	50/50	92
P2	14.93	85.53	50/50	92
P3	14.44	86.09	45.45/50	142
P3	14.54	86.1	45.45/51	142
P4	15.42	87.42	55/52.38	202
P4	15.44	87.52	55/52.38	202
P5	15.52	88.79	63.16/60	161
P5	15.49	88.8	63.16/60	161

Table S4. Final genome assembly of *T. marneffei* ATCC 18224

Contig name	Composition	Length	Coverage (X)
TMATCC001.1	Seq9, Seq8, Edge24, Seq2	6490868	381, 385, 685, 190
TMATCC002.1	Seq1	6416306	192
TMATCC003.1	Seq3	4186281	192
TMATCC004.1	Seq4	3673420	191
TMATCC005.1	Seq5	3245888	191
TMATCC006.1	Seq6	2022412	192
TMATCC007.1	Seq7	1827189	192
TMATCC008.1	Seq9, Seq8, Edge24	743805	381, 385, 685
TMATCC009.1	Seq10	180620	187
TMATCC010.1	Seq11	58913	186
TMATCC011.1	Seq12	26426	358

Table S5. 92 functional annotated genes in TMATCC008.1

Sequence ID	Start	End	Gene symbol	Description
TMatcc_010926	76790	78527	*SST2*	AMSH/STAMBP protein ubiquitin specific-protease
TMatcc_010943	116077	119015	*ADI1*	acireductone dioxygenase (Ni2+-requiring)
TMatcc_010946	122488	123728	*RAD14*	DNA repair protein
TMatcc_010948	125015	128632	*NOP2*	rRNA (cytosine-C5-)-methyltransferase
TMatcc_010950	130711	131871	*DYS1*	deoxyhypusine synthase
TMatcc_010957	153405	155072	*CWC27*	Peptidyl-prolyl isomerase
TMatcc_010960	161846	163201	*RFC3*	Replication Factor C Subunit3 (RFC3) proteins
TMatcc_010961	163584	164467	*ARC18*	Arc18p
TMatcc_010962	163789	166403	*TRM6*	tRNA (adenine(58)-N(1))-methyltransferase non-catalytic subunit trm6
TMatcc_010964	170286	173416	*CTA8*	Heat shock transcription factor
TMatcc_010971	186733	188634	*PIN4*	peptidylprolyl cis/trans isomerase, NIMA-interacting 4
TMatcc_010973	192274	195554	*PHO88*	phosphate transporter
TMatcc_010974	192274	193524	*HTZ1*	histone
TMatcc_010977	199311	202409	*HDA1*	Histone deacetylase hda1
TMatcc_010978	203318	208223	*STE23*	metalloprotease
TMatcc_010983	214045	214872	*CAB5*	putative dephospho-CoA kinase
TMatcc_010987	221714	223696	*ECM39*	alpha-1,6- mannosyltransferase
TMatcc_010992	236978	240909	*HTS1*	histidine--tRNA ligase
TMatcc_010993	239291	240884	*COQ6*	putative N,N-dimethylaniline monooxygenase
TMatcc_010998	248049	249798	*SNX4*	sorting nexin 4
TMatcc_011001	252599	255504	*KEX2*	pheromone processing endoprotease
TMatcc_011003	262715	264146	*TRR1*	thioredoxin-disulfide reductase
TMatcc_011006	266784	270370	*ALD6*	aldehyde dehydrogenase (NADP(+))
TMatcc_011008	272581	275028	*MDE1*	methylthioribulose 1-phosphate dehydratase
TMatcc_011009	275246	277242	*vps5*	sorting nexin 1
TMatcc_011018	295902	298838	*CCR4*	CCR4-NOT core exoribonuclease subunit
TMatcc_011021	302379	303688	*DCR2*	phosphoprotein phosphatase
TMatcc_011023	304306	307750	*DCR2*	phosphoprotein phosphatase
TMatcc_011026	314064	317585	*CCC2*	Cu(2+)-transporting P-type ATPase
TMatcc_011027	318333	320168	*rad1*	checkpoint clamp complex protein
TMatcc_011029	320294	322126	*GCN5*	histone acetyltransferase
TMatcc_011030	322290	325698	*RAX1*	transcription factor
TMatcc_011032	325877	330393	*RAD50*	MRX complex DNA-binding subunit
TMatcc_011035	332487	333841	*wc2*	white collar 2 type of transcription factor
TMatcc_011040	347800	350120	*sif3*	Sad1-interacting factor 3
TMatcc_011044	356408	357558	*COQ3*	ubiquinone biosynthesis O-methyltransferase, mitochondrial-like
TMatcc_011046	359838	363296	cta3	Calcium-transporting ATPase 3
TMatcc_011048	367631	379114	*RAM1*	protein farnesyltransferase
TMatcc_011049	367791	368708	*UBI4*	ubiquitin
TMatcc_011054	385660	386410	*YPD1*	Phosphorelay intermediate protein
TMatcc_011059	390865	392678	*AAT1*	aspartate transaminase
TMatcc_011063	398753	401196	*LYS21*	homocitrate synthase lys21
TMatcc_011064	401725	403617	*DPL1*	sphinganine-1-phosphate aldolase
TMatcc_011066	404036	404332	*MRPL33*	mitochondrial 54S ribosomal protein
TMatcc_011067	404968	407235	*SEC6*	SNARE-binding exocyst subunit
TMatcc_011071	413318	414041	*YFH1*	ferroxidase
TMatcc_011072	414537	415029	*DPH3*	Diphthamide biosynthesis protein 3
TMatcc_011074	416748	419660	*BDF1*	transcription initiation at TATA-containing promoter protein
TMatcc_011076	421133	425216	*UTP5*	Utp5p
TMatcc_011079	428539	429861	*BET4*	Rab geranylgeranyltransferase
TMatcc_011086	438616	441964	*EAF3*	Esa1p-associated factor
TMatcc_011088	443604	444491	*ISA1*	Iron-sulfur assembly protein 1
TMatcc_011090	446307	447331	*vti1*	v-SNARE protein
TMatcc_011091	447456	449212	*RVB1*	RuvB family ATP-dependent DNA helicase pontin
TMatcc_011094	450700	451955	*MNN11*	alpha-1,6-mannosyltransferase
TMatcc_011095	452085	453583	*TSC13*	3-oxo-5a-steroid 4- dehydrogenase
TMatcc_011096	453684	471664	*RPS6*	40S ribosomal protein eS6
TMatcc_011097	454923	467968	*TOM1*	E3 ubiquitin-protein ligase
TMatcc_011098	468084	471598	*PRS4*	ribose phosphate diphosphokinase subunit
TMatcc_011101	476879	479026	*RFG1*	slightly ste11-like protein
TMatcc_011104	485945	487017	*NRK1*	ribosylnicotinamide kinase
TMatcc_011105	487110	487909	*TXNL4A*	Thioredoxin-like 4A
TMatcc_011109	497831	500062	*PRM1*	pheromone-regulated protein
TMatcc_011116	518233	519151	*CDC31*	centrin
TMatcc_011117	519990	521594	*MGS1*	ssDNA-dependent ATPase
TMatcc_011119	524279	526334	*BNA3*	kynurenine--oxoglutarate transaminase
TMatcc_011122	534517	536824	*ACS2*	acetyl-coenzyme A synthetase 2

To be continued.

Sequence ID	Start	End	Gene symbol	Description
TMatcc_011125	547208	549021	*NDH51*	NADH dehydrogenase [ubiquinone] flavoprotein 1, mitochond rial
TMatcc_011127	552096	553073	*SIT4*	type 2A-related serine/threonine-protein phosphatase
TMatcc_011128	553204	556950	*SIT4*	type 2A-related serine/threonine-protein phosphatase
TMatcc_011131	557498	567234	*SMF3*	NRAMP-like transporter
TMatcc_011130	557498	558514	*VPS60*	Vps60p
TMatcc_011138	576037	585039	*BLM3*	Proteasome activator
TMatcc_011139	577224	578746	*POT1*	acetyl-CoA C-acyltransferase
TMatcc_011141	586127	587677	*GPI18*	ER membrane glycoprotein subunit of the GPI transamidase co mplex-like protein
TMatcc_011151	610303	613673	*SAS3*	Histone acetyltransferase
TMatcc_011155	617271	620409	*VPH1*	H(+)-transporting V0 sector ATPase subunit a
TMatcc_011156	617271	624918	*FBP1*	fructose 1,6-bisphosphate 1-phosphatase
TMatcc_011159	630554	634120	*AHA1*	Co-chaperone
TMatcc_011160	632758	634444	*LYS9*	saccharopine dehydrogenase (NADP+, L-glutamate-forming)
TMatcc_011161	635098	636974	*RTG2*	retrograde regulation protein 2
TMatcc_011162	637380	638617	*RER2*	ditrans,polycis-polyprenyl diphosphate synthase
TMatcc_011164	639163	641706	*SCT1*	Glycerol-3-phosphate/dihydroxyacetone phosphate acyltransferase
TMatcc_011169	649261	650102	*ENDOGLUCANASE1*	endoglucanase-1
TMatcc_011173	659120	662219	*CDC24*	Guanine nucleotide exchange factor for Cdc42p
TMatcc_011179	672018	674796	*SOD2*	superoxide dismutase
TMatcc_011180	673844	675199	*MPG1*	mannose-1-phosphate guanyltransferase
TMatcc_011181	675196	682412	*YME1*	i-AAA protease
TMatcc_011184	687896	693457	*GUT1*	glycerol kinase
TMatcc_011186	694524	704133	*VPS13*	Vacuolar protein sorting-associated protein 13
TMatcc_011189	725343	726801	*ERG27*	3-keto-steroid reductase
TMatcc_011192	731201	732374	*PSF3*	DNA replication protein

Table S6. GO and KEGG enrichment results of TMATCC008.1

Category	Term	Count	*P* Value	Genes	Fold Enrichment
			**Molecular Function**		
GOTERM_MF_DIRECT	GO:0046872~metal ion binding	38	4.84E-04	*TCB1, YD23B, CYB2, PYP1, CP51, ZAP1, RAD50, ETP1, SMF1, ULS1, AZF1, GAT2, SODM, RAD14, PUR6, PIP2, SAS3, MTNB, CDC31, CCR4, ISA1, NRK1, CAN, ALN, ATU2, YME1, STE23, KEX2, RRAAH, KPR4, POLH, WRIP1, DPH3, ATN2, ARGR2, F16P, FNTB, HOSM*	1.736923341
GOTERM_MF_DIRECT	GO:0016491~oxidoreductase activity	18	8.96E-04	*FMS1, CYB2, TECR, G3P1, TRXB2, CP51, LYS9, COQ6, YL460, FRDA, ALDH5, YM94, GTO2, AAD4, DPH3, YJ66, SODM, ERG27*	2.438980012
GOTERM_MF_DIRECT	GO:0070577~lysine-acetylated histone binding	3	1.30E-02	*BDF1, GCN5, BLM10*	16.3179377
GOTERM_MF_DIRECT	GO:0022857~transmembrane transporter activity	9	0.025876	*MAL11, QDR2, VBA5, DIP5, TNA1, TPO1, HNM1, MCH5, GIT1*	2.501289721
GOTERM_MF_DIRECT	GO:0000009~alpha-1,6-mannosyltransferase activity	3	0.02646	*ALG12, GPI18, MNN11*	11.42255639
GOTERM_MF_DIRECT	GO:0004659~prenyltransferase activity	3	0.031795	*RER2, FNTB, PGTA*	10.38414217
GOTERM_MF_DIRECT	GO:0016740~transferase activity	28	0.049164	*YD23B, THIK, GCN5, RKM1, GPT1, GLPK, GPI18, GTO2, CAB5, DHYS, BNA3, MNN11, AATC, PGTA, ALG12, SAS3, TOM1, EAF3, COQ3, NRK1, CEM1, NOP2, KPR4, POLH, RER2, FNTB, HOSM, MPG1*	1.41393271
GOTERM_MF_DIRECT	GO:0034986~iron chaperone activity	2	0.051458	*DPH3, FRDA*	38.07518797
GOTERM_MF_DIRECT	GO:0008270~zinc ion binding	23	0.053696	*PIP2, YD23B, HDA1, SAS3, MTNB, ZAP1, RAD50, CAN, ETP1, ALN, YME1, STE23, ULS1, AZF1, POLH, WRIP1, DPH3, UBP13, GAT2, ARGR2, RAD14, FNTB, HOSM*	1.476777948
			**Cellular Component**		
GOTERM_CC_DIRECT	GO:0005739~mitochondrion	41	9.64E-03	*TCB1, TECR, CYB2, THIK, MGLL, TNA1, RM33, RAD50, PTH2, RM24, ULS1, YG5L, ALDH5, GLPK, GPI18, CAB5, QCR6, SODM, BNA3, MPC1, G3P1, TRXB2, COQ3, ISA1, HSF, CAN, COQ6, RM36, YME1, STE23, CEM1, FRDA, POLH, ARPC3, ATN2, VPS13, PHO88, SYH, SA185, HOSM, RMD8*	1.448619002
GOTERM_CC_DIRECT	GO:0005886~plasma membrane	20	4.47E-02	*TCB1, MAL11, MGLL, DIP5, GYP5, G3P1, YGI2, SEC3, SST2, MCH5, PRM1, SMF1, RAX1, YG5L, RSN1, QDR2, VBA5, TPO1, ATN2, GIT1*	1.57804158
GOTERM_CC_DIRECT	GO:0005789~endoplasmic reticulum membrane	16	4.78E-02	*TCB1, DSC3, ALG12, TECR, FAR8, NNF2, CP51, GPT1, YOS1, GPI18, SGPL, VTI1, RER2, PHO88, CALX, ERG27*	1.690428932
GOTERM_CC_DIRECT	GO:0005783~endoplasmic reticulum	23	0.054105	*TCB1, DSC3, ALG12, TECR, MGLL, CYPB, UBP15, FAR8, NNF2, CP51, GPT1, YOS1, GPI18, RSN1, SGPL, CAB5, HNM1, RER2, PHO88, CALX, MNN11, ERG27, RMD8*	1.480381206
GOTERM_CC_DIRECT	GO:0005811~lipid particle	5	0.058489	*MGLL, G3P1, CAB5, RER2, ERG27*	3.384159483
			**Biological Process**		
GOTERM_BP_DIRECT	GO:0009267~cellular response to starvation	4	1.32E-02	*SGPL, VPS13, HOS4, SNT1*	7.857931034
GOTERM_BP_DIRECT	GO:0006357~regulation of transcription from RNA polymerase II promoter	13	2.57E-02	*SAS3, EAF3, ZAP1, RTG2, CCR4, GCN5, HSF, MET30, AZF1, RUVB1, GAT2, H2AZ, SNT1*	2.018836037
GOTERM_BP_DIRECT	GO:0035753~maintenance of DNA trinucleotide repeats	3	0.030051	*RTG2, RAD50, CTF8*	10.7153605
GOTERM_BP_DIRECT	GO:0060003~copper ion export	2	0.049918	*QDR2, ATU2*	39.28965517
GOTERM_BP_DIRECT	GO:0006260~DNA replication	6	0.055719	*PSF3, RFC3, POLH, WRIP1, CCR4, CTF8*	2.874852817
GOTERM_BP_DIRECT	GO:0055085~transmembrane transport	12	0.058379	*MAL11, QDR2, VBA5, DIP5, TNA1, TPO1, ATN2, HNM1, MCH5, SMF1, ATU2, GIT1*	1.841702586
GOTERM_BP_DIRECT	GO:0006338~chromatin remodeling	8	0.063415	*HDA1, EAF3, RUVB1, BDF1, HOS4, GCN5, ULS1, H2AZ*	2.245123153
GOTERM_BP_DIRECT	GO:0006355~regulation of transcription, DNA-templated	11	0.071351	*PIP2, HDA1, SAS3, EAF3, RUVB1, BDF1, GAT2, ARGR2, HSF, SNT1, H2AZ*	1.846949602
GOTERM_BP_DIRECT	GO:0010995~free ubiquitin chain depolymerization	2	0.073941	*UBP15, UBP13*	26.19310345
GOTERM_BP_DIRECT	GO:0045944~positive regulation of transcription from RNA polymerase II promoter	12	0.074123	*FMS1, PIP2, HDA1, AZF1, SAS3, CDC31, ZAP1, RTG2, ROX1, GCN5, ARGR2, HSF*	1.765827199
GOTERM_BP_DIRECT	GO:0006631~fatty acid metabolic process	4	0.079309	*PIP2, TECR, THIK, CEM1*	3.928965517
GOTERM_BP_DIRECT	GO:0006974~cellular response to DNA damage stimulus	10	0.095436	*EAF3, POLH, RUVB1, WRIP1, BDF1, HOS4, BLM10, RAD50, RAD14, SNT1*	1.818965517
GOTERM_BP_DIRECT	GO:0072593~reactive oxygen species metabolic process	2	0.097361	*F16P, SODM*	19.64482759
GOTERM_BP_DIRECT	GO:0051601~exocyst localization	2	0.097361	*SEC6, SEC3*	19.64482759
			**KEGG enrichment**		
KEGG_PATHWAY	sce01110:Biosynthesis of secondary metabolites	19	0.009251	*TECR, THIK, G3P1, CP51, COQ3, LYS9, COQ6, GPT1, ACS2, ALDH5, KPR4, RER2, F16P, PUR6, FNTB, ERG27, HOSM, AATC, MPG1*	1.838972798
KEGG_PATHWAY	sce01100:Metabolic pathways	34	0.009697	*TECR, CYB2, THIK, MGLL, CP51, VPP1, ACS2, GPT1, ALDH5, GLPK, GPI18, SGPL, CAB5, QCR6, BNA3, PUR6, MNN11, ERG27, AATC, ALG12, FMS1, G3P1, MTNB, COQ3, LYS9, NRK1, CAN, COQ6, ALN, CEM1, KPR4, F16P, HOSM, MPG1*	1.447454252
KEGG_PATHWAY	sce00561:Glycerolipid metabolism	4	0.063807	*ALDH5, GLPK, MGLL, GPT1*	4.246575342

Table S7. GO enrichment results of the DEGs in TMATCC008.1

Category	Term	Count	*P* Value	Genes	Fold Enrichment
GOTERM_BP_DIRECT	GO:0055085~transmembrane transport	5	0.023	*VBA5, TPO1, ENA2, HNM1, MCH5*	4.401
GOTERM_CC_DIRECT	GO:0005886~plasma membrane	9	0.004	*TCB1, RSN1, VBA5, TPO1, ENA2, SEC3, MCH5, PRM1, SMF1*	3.191
GOTERM_CC_DIRECT	GO:0031390~Ctf18 RFC-like complex	2	0.032	*RFC3, CTF8*	59.866
GOTERM_MF_DIRECT	GO:0003677~DNA binding	8	0.007	*TY2B-DR3, RFC3, ROX1, GAT2, ARG81, RAD14, GAL80, CTF8*	3.321
GOTERM_MF_DIRECT	GO:0046872~metal ion binding	10	0.015	*TCB1, TY2B-DR3, CYB2, ENA2, GAT2, ARG81, FBP1, RAD14, DAL1, SMF1*	2.383
GOTERM_MF_DIRECT	GO:0022857~transmembrane transporter activity	4	0.024	*VBA5, TPO1, HNM1, MCH5*	6.182
GOTERM_MF_DIRECT	GO:0016491~oxidoreductase activity	5	0.032	*FMS1, CYB2, YMR315W, ECM4, AAD4*	3.955
GOTERM_MF_DIRECT	GO:0046873~metal ion transmembrane transporter activity	2	0.0426	*ENA2, SMF1*	44.646

Table S8. Repeat contents of six *T. marneffei* strains

Strain ID	PM1	TM4	11CN-03-130	GZ8H79	11CN-20-091	ATCC 18224
Repeat content	5.24%	5.21%	5.48%	5.82%	5.00%	5.05%
SINEs	0	0	0	0	0	0
LINEs	0.86%	0.72%	0.79%	1.02%	1.21%	0.59%
LTR elements	0.85%	1.07%	1.33%	1.33%	0.73%	1.46%
DNA elements	1.11%	1.04%	0.67%	0.91%	0.85%	1.07%
Total interspersed repeats	4.18%	4.14%	4.07%	4.51%	3.81%	3.89%
Small RNA	0.13%	0.01%	0.39%	0.01%	0.17%	0.01%
Satellites	0	0	0.01 %	0	0	0
Simple repeats	0.93 %	0.93 %	0.90 %	1.19 %	0.91 %	1.03 %
Low complexity	0.11 %	0.12 %	0.11 %	0.11 %	0.11 %	0.12 %

Table S9. Overview statistics of LTR retrotransposon ORFs in six strains

Strain ID	PM1	TM4	11CN-03-130	GZ8H79	11CN-20-091	ATCC 18224	Total
**Ty1/*Copia***							
RH & RT & INT	24	20	8	27	11	30	120
RH & RT	5	5	12	10	13	17	62
RT	3	5	14	6	1	3	32
Total	32	30	34	43	25	50	214
**Ty3/*Gypsy***							
RH & RT & INT	0	3	2	0	0	3	8
RH & RT	4	5	6	6	15	6	42
RT	6	4	7	10	5	5	37
Total	10	12	15	16	20	14	87

Table S10. Expression levels of two clusters of retrotransposons

Cluster	Temperature	$\log_{10}(TPM+1)$	Reads number
Cluster A	25 °C	3.86	705
		3.91	644
	37 °C	0	0
		0	0
Cluster B	25 °C	4.42	3382
		4.48	3224
	37 °C	4.62	6361
		4.69	6807
